# The impact of slow mobility and built environment characteristics on 12.5-year all-cause mortality among older women and men: A prospective cohort study from Poland

**DOI:** 10.1016/j.ssmph.2025.101841

**Published:** 2025-07-11

**Authors:** Katarzyna Zawisza, Michalina Gajdzica, Alberto Raggi, Beata Tobiasz-Adamczyk

**Affiliations:** a Jagiellonian University Medical College, Chair of Epidemiology and Preventive Medicine, Department of Epidemiology, Krakow, Poland; b Jagiellonian University Medical College, Chair of Epidemiology and Preventive Medicine, Department of Medical Sociology, Krakow, Poland; c Neurology, Public Health and Disability Unit, Fondazione IRCCS Istituto Neurologico Carlo Besta, Milan, Italy

**Keywords:** Slow mobility, Older women and men, Built environment, All-cause mortality

## Abstract

Worldwide initiatives promoting mobility modes such as walking or cycling as low-cost and zero-emission forms of transport, have highlighted the possible health benefits of slow mobility. Identifying crucial elements of the built environment (BE) for slow mobility users, especially older adults, is important.

The study aimed to: compare the mortality risk of slow mode users with other transport mode users in both men and women; verify, which aspects of subjective and objective assessment of the BE are relevant as risk factors of 12.5-year all-cause mortality across various mobility mode users; examine social participation as a mediator of the relationship.

The Polish part of the COURAGE in Europe cross-sectional baseline study was conducted in 2011. The analysis included 1166 face-to-face interviews with randomly selected community-dwelling individuals aged 65 years or older from Poland. Information about deaths was obtained from the State Systems Department on Mar 1, 2024. The outdoor BE was assessed by trained interviewers’ direct observations. The Cox proportional hazard models were used.

Higher quality of streetscapes (in women) and walkways (in men) for those who mainly walk in their neighborhood, and bikeways (in women who cycle) were found to be significant protective factors against mortality. Walkways and bikeways were associated with a higher risk of death in the fast mode of transportation group of men. Mediation effect of social participation was found in unadjusted models.

The findings underline the importance of planning and organizing the BE from an age–friendly perspective and the need for a holistic approach to urban planning.

## Highlights

• Objective assessment of streetscape is a predictor of all-cause mortality in slow mobility users (in women).
•Objective assessment of walkways (in men) is a predictor of all-cause mortality in slow mobility users.
•Objective assessment of bikeways is a predictor of all-cause mortality in women who cycle.
•Walkways and bikeways were associated with a higher risk of death in the fast mode of transportation group of men.
•Mediation effects of formal or informal participation were found in unadjusted model.

## 1 Introduction

Since the 1980s, when the concept of healthy and active ageing first emerged, scientists have focused on the everyday activities of older adults. Mobility is a key aspect of daily activity and health-related quality of life of older people, as well as an important indicator of active and healthy ageing. Webber et al. (2010) define mobility as "the ability to move oneself (either independently or with the use of assistive devices or transportation) within community environments that extend from one's home, to the neighborhood and to regions beyond". The mobility provides psychosocial benefits for older adults, such as maintaining social relationships with both peers and younger generations, participating in neighborhood social capital achievements (Kawachi & Berkman, 2000) by accessing several services, and involvement in existing informal and formal social networks. A socially cohesive neighborhood can significantly enhance the well-being of older adults, promote personal safety and the ability to participate in the creation of an appropriate, socially trusting environment (Kawachi & Berkman, 2000).

Focusing on mobility in older adults requires defining the life space in which they move, considering both their physical and social environment (Seinsche et al., 2023). The social dimension of mobility in later life refers to older adults’ ability to maintain or establish new social relationships in shared spaces. Mobility strengthens their sense of integration within their social environment and, in turn, enhances positive feelings and emotions. The symbolic visibility of older adults in the environment increases alongside broad social changes (increasing number of older people in societies and awareness of the ageing process, increases positive attitudes towards older people, counteracting ageism) (Joseph et al., 2006). Mobility which is associated with going outside the home environment to a broader neighborhood environment, promotes autonomy and social participation in older age (Lindström & Rosvall, 2019).

Research indicated that walking, driving and public transport were the main forms of mobility for older adults around their neighborhood (Rantanen et al., 2012). Walking in a familiar environment was the most common physical activity for older adults (Czarnecki et al., 2020) and had positive effects on health, cognition and well-being (Kramer & Erickson, 2007; Lee & Buchner, 2008). However, walking can be risky as it exposes individuals to the possibility of accidents and falls (Tournier et al., 2016).

Mobility limitation - defined as either a performance deficit assessed by an objective mobility test or perceived difficulty in mobility - increases with age and is often the first sign of further functional decline. Mobility limitations affect the ability to perform daily tasks and may lead to the need for assistance, as well as an increased risk of disability (Rantakokko et al., 2015). Older adults with mobility limitations also had a lower health-related quality of life (Groessl et al., 2019) and this relationship can be mediated by environmental features.

Webber et al. (2010) developed a theoretical framework that illustrates the concept of mobility through five fundamental categories of determinants: cognitive, psychosocial, physical, environmental, and financial. Additionally, gender, culture, and personal life history are conceptualized as critical crosscutting influences. The model suggests that “research needs to be more interdisciplinary, current mobility findings should be interpreted more comprehensively, and new, more complex strategies should be developed to address mobility concerns“ (Webber et al., 2010).

The life course of mobility provides an opportunity to observe changes in gait, balance and strength. Age-related physical changes associated with mobility include physical, cognitive, neuromuscular and behavioral factors, whereas environmental factors include accessibility (distance), perceived environmental safety and walkability (Freiberger et al., 2020). Walking difficulties may start to develop in midlife and become more prevalent with age (Rantanen et al., 2012). A study involving community-dwelling older adults (75–90 years, n = 848) explored perceived environmental barriers and facilitators to outdoor mobility (Rantakokko et al., 2015). It found that 41 % of participants had restricted life-space. Those who reported one or more environmental barriers had more than double the odds of restricted life-space compared to those who reported no barriers, even after adjustment for health, functioning and socio-economic status (Rantakokko et al., 2015). Previous studies have shown that perceiving the neighborhood environment as more accessible is the most relevant factor associated with mobility limitations (Raggi et al., 2018).

The European Commission, among other worldwide initiatives, promotes low-cost and zero-emission mobility modes such as walking or cycling (slow modes of transportation) in the New European Urban Mobility Framework, with potential benefits in preventing health problems (EU, 2021). Parallel to this, it is important to assess the built environment from an age–friendly perspective, i.e. ensuring that the elements of the built environment (BE) are suitable for slow mobility users (those who mainly walk or cycle in their neighborhood), most of whom are older adults with lower functional status.

### 1.1 Mobility/mobility limitations as a predictor of mortality

The relationship between mobility disability and mortality is well established. Over a 10-year period, all-cause mortality in older people in San Paulo was defined by mobility status, confirming that limitations in walking speed and gait speed are predictors of mortality (do Nascimento et al., 2022). Among older Spanish adults aged over 70, frailty was associated with increased mortality. Additionally, it was linked to limitations in activities of daily living (ADL) and reduced mobility (Abizanda et al., 2013). A Japanese study involving 874 community-dwelling healthy older adults, found that the TUG time (timed up and go) test was associated with all-cause mortality (Otsuka et al., 2021). In a cohort of 3075 community-dwelling adults aged 70–79, the ability to complete the long-distance corridor walk and total performance time were important prognostic factors for all-cause mortality and mobility disability over 4.9 years (Newman et al., 2006).

A prospective cohort study of 1280 older adults with persistent mobility limitations (walking, climbing stairs, getting out of chairs and ADL) who did not report restriction in ADL, showed that the risk of death was significantly higher at both 2 and 4 years of follow-up compared to subjects with no difficulties in mobility and ADL (Khokhar et al., 2001). Kennedy et al. (2017) showed that life-space scores (i.e., self-reported assessment of mobility in the community, from one's room to outside the town) and changes in life-space were significantly associated with the risk of death over a 6-month period. Another study found that life-space mobility over a one month period, predicted mortality in older men (Mackey et al., 2014). Finally, energy decline was a common and significant predictor of disability risk and mortality in a group of 2021 participants free of mobility disability (Sprague et al., 2021).

### 1.2 Built environment characteristics as predictors of mortality

The literature on the association between BE and mortality is inconsistent. Some studies have shown that the presence of walkable green spaces decreases the risk of mortality (Rojas-Rueda et al., 2019; Takano et al., 2002), whereas other research suggest that living in commercial or residential areas is associated with lower all-cause mortality compared to living near larger green space (Lin et al., 2021). This has been explained by the fact that these types of areas are more accessible destinations within walking distance from home, which encourages walking as a means of transportation. A systematic review underscores that safe, walkable, and aesthetically pleasing neighborhoods with access to destinations and services are positively associated with health-enhancing levels of physical activity (Barnett et al., 2017)

A reduction of all-cause mortality through building bicycle lanes and paths along busy roads with mixed traffic was estimated by Schepers et al. (2015). The literature review showed that purpose-built bicycle only facilities (e.g. bike lanes) reduce the risk of crashes and injuries compared to cycling on-road with traffic or offroad with pedestrians (Reynolds et al., 2009). Some researchers argue that building physically separated bicycle lanes may be associated with worse visibility and conflicts between different road users, potentially leading to poorer road safety outcomes (Schepers et al., 2015).

### 1.3 Social participation as a mediator of the association between BE characteristics and health

The built environment can play a significant role in enhancing older adults' sense of inclusion (Scharlach & Lehning, 2013). Among other effects, it may foster social participation and contribute to their overall well-being. Social participation is a known factor influencing mortality (Zawisza et al., 2024), and it is also highlighted as benefit of the ability to move independently (Kawachi & Berkman, 2000). The previous study showed that higher degree of acceptance of the leisure environment and the landscape environment was associated with health of older people through the intermediary role of outdoor exercise and social participation (Zheng et al., 2022). There is a lack of studies examining social inclusion, particularly social participation, as a mediating factor in the association between BE and mortality. Previous research has shown that residential environment is associated with older adults’ health, particularly through social activities (Liu et al., 2017). Similarly, another study found that social support, beyond walking time and depression, mediated the relationship between built environment and frailty (Mori et al., 2023).

When addressing initiatives that promote slow mobility modes, it is important to verify their association with mortality. In fact, while some features of the built environment are predictors of mobility or health-related quality of life, less is known about these characteristics as predictors of mortality. Since different characteristics of the built environment appear to be important for different mode users, subgroup analysis across transportation mode is crucial. Understanding the determinants of mortality related to facilities and barriers of built environment is important for public health policy and urban planning. Specifically, for older adults who mainly walk or cycle. Finally, there is a need to focus on facilities that facilitate face-to-face social interactions and foster social participation within this age group in their neighborhood, as these may help decrease the risk of mortality.

## 2 The aims of the study

The aims of the study were 1) to compare the mortality risk of slow mobility users with that of other transport mode users in both men and women; 2) to verify which aspects of subjective and objective assessment of the built environment are relevant as risk factors for 12.5-year all-cause mortality among slow mobility users compared to other transport mode users; 3) to examine whether social participation mediates the relationship between built environment characteristics and survival time.

## 3 Methods

### 3.1 Study design and participants

Data come from a prospective cohort study conducted in Poland. The baseline information was gathered in 2011 in cross-sectional survey as a part of the COURAGE *in Europe* study (Leonardi et al., 2014). Participants were randomly selected from the non-institutionalized Polish adult population (n = 4071) based on the multistage clustered design with an oversampling of people aged 50–79 years and >80 years. The strata were created depending on age and the geographical administrative regions and catchment area size. The response rate was 66.5 %. Face-to-face interviews were performed at the respondents’ homes using computer-assisted personal interviewing (CAPI). Additionally, the outdoor built environment was assessed through direct observations by specially trained interviewers.

After excluding people aged below 65 years and proxy respondents, and those who do not move autonomously in their neighborhood, the final analyzed sample included 473 men and 693 women aged 65 years or older (see Supplementary Fig. S2).

All data were weighted to account for the sampling design and to generalize the study sample to the reference population. Normalized weights were used. Post-stratification corrections were made to the weights to adjust for the population distribution obtained from the national census, and for non-response.

## 4 Measures

### 4.1 Mortality

In order to verify the vital status and date of deaths (on Mar 1, 2024) we sent to the State System Department, Digital Affairs – Chancellery of the Prime Minister of Poland database including ID of respondents, names, surnames and personal identification numbers.

### 4.2 Mobility mode

Mobility mode was assessed by the question “How do you usually move around *autonomously* in your neighborhood?“, where respondents indicated maximum two of the following response options 1. “walking”, 2. “walking with dog”, 3. “using means of public transportation”, 4. “using private motor vehicle (s)”, 5. “cycling”, 6. “using personal mobility aid(s) (using a wheelchair, a rollator, a white cane, crutches, etc)” and 9. “I don't move around autonomously in my neighborhood”. It was decided to distinguish two group of slow mobility users (those who mainly walk or cycle in their neighborhood) from the rest of the respondents (fast mobility group). Since there was a quite large group of respondents who cycle, we decided to extract these individuals from the rest of slow mode of transportation users. We made this decision also because of different characteristics of the built environment, e.g., walkways or bikeways, which seemed to be important for walkers and cyclists. Their perception of BE features may be also various form fast mode transportation users. Thus, participants were gathered into the following three categories: “slow mobility users - walkers” in case of pointed only options 1., 2. or 6.; “slow mobility users - cyclists” if they reported option 5. as one of two; and “fast mobility users”, when options 3. or 4. were reported (but not 5.). In case of the fourth group (if the last 9. category was reported), they relied on other people for support, aid or care and there was no further information on which mode of transportation they used with assistance, if any. This group was excluded from the analyses.

### 4.3 Built environment

Courage Built Environment Self-Reported Questionnaire (CBE-SR) comprises 19 items grouped into four indexes: “Usability of the neighborhood environment” (e.g. presence of useful places such as shops, banks, health centers; presence of community or leisure facilities, bus stops), “Hindrance of walkable environment” (e.g presence of discontinuous, too narrow or blocked by obstacles walkways, inadequate street light), “Easiness of use of public buildings, places and facilities” (e.g. easiness to move through internal pathways, presence of signs in buildings that give essential, simple and clear information) “Usability of the living place” (e.g. presence of comfortable to use: bathroom, stairs at entrance or stairs connected different levels). For each of the four scales, scores range between 0 and 100, higher scores indicate more favorable conditions and address, respectively, a neighborhood environment perceived as more useable, walkable environment perceived as less hindering (reversed items), public buildings, places and facilities perceived as more easy to use, and living place perceived as less risky and more useable. The tool was validated previously (Raggi et al., 2014).

The COURAGE Built Environment Outdoor Checklist (CBE-OUT) is the measurement, which objectively evaluate of the technical measurements of features of the environment in the respondents’ surrounding neighborhood. The diameter of this circular space is equivalent to 1 km. It is composed of 128 items. Interviewers marked presence of elements of built environment that are not only *on* the road but also *visible from* the road. The following indices were developed: Streetscape (e.g. number of lines road, condition of road surface, presence of lights, transit stops, traffic calming devices, trees), Walkways (e.g. presence, wide, slope, quality, cleanliness, presence of construction sites), Bikeways (presence and continuous), Street crossing/intersections (e.g. White or colored painted line (s), different road surface paving), Parking facilities, Public facilities and features of the street, Land-use visible along the street/road (e.g. multi-family homes, open-to-public green spaces, commercial and industrial buildings), Site decay/urban blight (e.g. litter, abandoned cars and buildings, animal droppings). Its score ranges from 0 to 100, where higher score means more positive impact on the accessibility of neighborhood for healthy aging. The detailed list of items is available in Quintas et al. (2014).

### 4.4 Measurement of other included variables

Age (in years) was calculated as the difference between the date of the interview and the date of birth. Marital status (married or cohabiting vs. other, including never married, not cohabiting, separated, divorced, and widowed), level of education (primary, vocational, high school, and university), and place of living (rural vs. urban) were self-reported.

Health-related behaviors: Smoking status was categorized into three groups: current, past and never smokers. Alcohol consumption patterns were assessed in three groups: 1. high risk drinkers (consumers of at least five (in men) or four (in women) standard alcoholic drinks per day on at least 1 day during the week before the interview), 2. low risk drinkers, and 3. lifetime abstainers. Body mass index (BMI) was calculated as weight (kg) divided by height (m^2^), where weight was measured using a calibrated electronic weighing scale, and height was measured using a stadiometer. Physical activity was measured with the Global Physical Activity Questionnaire (GPAQ v2), that differentiates between work and leisure, recreational and sport-related activities, and records how many days and for how long (minutes or hours) each activity was undertaken in the past week. It considers the intensity of activity defined as Metabolic Equivalent to Task (MET). The levels of physical activity were created using conventional cut-off points: high, moderate and low.

Health and functioning status: Grip strength was measured using a Smedley's hand dynamometer. The total number of diseases was measured by self-reported data about the diagnosis of the following conditions: arthritis, angina, stroke, diabetes, COPD, asthma, cataracts, depression, dental problems. Visual impairment was assessed with the question “In the past 30 days, how much difficulty have you had seeing and recognizing objects and familiar people from a distance of about 20 m (e.g., across the street)?”. Hearing impairment was measured using the question ”In the past 30 days, how much difficulty have you had hearing and recognizing sounds from a similar distance?”. Responses were rated on a 5-point scale, from 1—no difficulty to 5—extreme difficulty. The presence or absence of difficulties in independent living was assessed in terms of activities of daily living (ADL), such as eating, bathing, and dressing, as well as more complex instrumental activities of daily living (IADL), such as using transportation, housekeeping, and preparing food. Difficulties were considered present if the person answered “severe”, “extreme/cannot do it” to any of the questions. Walking test required participants to walk a distance of 4 m at a quick pace, with the time taken recorded in seconds.

Perceived safety on the street was measured using the question “How safe do you feel walking alone on the street (in your neighborhood) after dark?”. A 5-point response scale was used, ranging from 1—completely safe to 5—not safe at all.

#### 4.4.1 Psychosocial factors

Loneliness was assessed by means of the Three-item UCLA Loneliness Scale (Hughes et al., 2004). The items were related to frequency of feeling of lack companionship, left out and isolated from others. A 3-point response scale was used (hardly ever; some of the time; often).

The level of social networks was measured by the COURAGE Social 10.13039/100031212Network Index (COURAGE-SNI) which assesses elements of function of social networks (frequency of direct contact, ties and social support) in eight structural components (spouse or partner, parents, children, grandchildren, other relatives, neighbors, friends and co-workers) (Zawisza et al., 2014). The perceived social support was measured by the OSLO-3 Social Support Scale (Dalgard, 1996). The aforementioned scales were transformed to a 0–100 point scale using a unitarization method. Higher scores indicated greater level of loneliness, higher level of social network and stronger social support.

Trust in neighbors – measured using the item *First, think about people in your neighborhood. Generally speaking, would you say that you can trust them?* with the five-point Likert response scale ranging from *to a very great extent* to *a very small extent.*

The informal and formal social participation scales each consist of four items. The former concerns e.g. having friends over at the home, visiting or hosting someone form a different neighborhood, and socializing with co-workers outside of work. The later includes items related to attending group or organizational meeting, participating in public meetings, and working with people from one's neighborhood to fix or improve something (Zawisza et al., 2024). The scales ranged from 0 to 100 points and higher values were interpreted as higher level of social participation. Additionality, the item related to frequency of religious services attendance with five-point response scale ranging from *never* to *daily*.

### 4.5 Statistical analysis

The comparison of the characteristics of men and women across different mobility modes (Table 1) was conducted using the chi-square test or the Kruskal-Wallis test.

**Table 1:** Baseline characteristics across mobility mode of men and women aged 65+, Polish part of the COURAGE in Europe study (N = 1166), 2011. Weighted data.

		Men				Women			
		Slow mobility users		Fast mobility users (n = 83) 18.9 %	*p value*	Slow mobility users		Fast mobility users (n = 85) 13.3 %	*p value*
		Walkers (n = 292) 60.7 %	Cyclists (n = 98) 20.4 %			Walkers (n = 524) 77.2 %	Cyclists (n = 84) 9.5 %		
**Mortality** (%)	Alive	47.6	51.1	48.3	*0.851*	60.5	75.3	69.2	*0.032*
	Deceased	52.4	48.9	51.7		39.5	24.7	30.8	
**Age** [Median (Q1; Q3)]		72 (68; 78)	73 (69; 76)	73 (69; 78)	*0.731*	74 (69; 80)	69 (66; 72)	74 (69; 81)	*<0.001*
**Married or cohabited** (%)		79.4	89.9	85.3	*0.061*	42.9	60.8	48.3	*0.027*
**Level of education** (%)	Priamary	31.8	43.9	14.1	*0.001*	51.4	48.4	38.0	*0.421*
	Vocational	28.0	28.5	20.7		14.1	14.8	17.8	
	High school	23.5	13.7	37.3		23.6	22.1	31.6	
	University	16.7	13.9	28.0		11.0	14.7	12.6	
**Urban place of living** (%)		69.9	42.6	76.7	*<0.001*	70.8	33.8	77.7	*<0.001*
**Smoking** (%)	Never smoked	28.1	29.1	25.1	*0.222*	74.6	82.0	71.6	*0.507*
	Current smoker	28.3	17.8	21.2		6.9	5.2	10.3	
	Not current smoker	43.6	53.1	53.7		18.6	12.8	18.1	
**Alcohol consumption** (%)	Lifetime abstainers	5.0	5.2	7.4	*0.189*	27.8	26.8	25.9	*0.981*
	Low risk drinkers	80.8	83.7	88.5		71.1	72.0	73.2	
	Infrequent high risk drinkers	14.2	11.1	4.1		1.1	1.3	0.9	
**BMI** [Median (Q1; Q3)]		27.9 (24.7; 30.4)	28.7 (24.9; 31.9)	30.1 (27.7; 31.2)	*<0.001*	28.8 (25.7; 32.4)	29.8 (25.5; 33.5)	28.6 (26.0; 32.9)	*0.932*
**Physical activity** (%)	High	42.4	54.9	42.5	*0.073*	40.7	64.3	51.2	*<0.001*
	Moderate	17.0	21.0	21.9		22.8	23.5	21.6	
	Low	40.6	24.1	35.6		36.6	12.2	27.2	
**Grip strength** (%)	Normal	71.8	77.3	76.5	*0.548*	59.9	84.9	66.4	*<0.001*
**Total number of diseases** [Median (Q1; Q3) [Mean (SD)]]		1 (0; 2) [1.2 (1.1)]	1 (0; 2) [1.2 (1.2)]	1 (0; 2) [1.3 (1.4)]	*0.958*	1 (1; 2) [1.6 (1.3)]	1 (0; 2) [1.2 (1.2)]	1 (1; 2) [1.5 (1.3)]	*0.020*
**Visual difficulty** (%)	None	64.8	76.1	73.7	*0.006*	58.2	65.1	68.7	*0.100*
	Mild or moderate	29.3	13.2	24.5		33.7	32.5	22.6	
	Severe or extreme	5.9	10.7	1.8		8.1	2.4	8.7	
**Hearing difficulty** (%)	None	63.0	51.4	55.1	*0.289*	66.4	74.3	60.2	*0.443*
	Mild or moderate	30.6	41.3	37.1		28.2	24.0	33.5	
	Severe or extreme	6.4	7.3	7.7		5.4	1.6	6.3	
**ADL scale** [Median (Q1; Q3) [Mean (SD)]]		0 (0; 2) [1.8 (3.2)]	0 (0; 3) [1.8 (2.9)]	0 (0; 2) [1.6 (2.7)]	*0.906*	1 (0; 6) [3.3 (4.0)]	0 (0; 2) [1.7 (2.9)]	0 (0; 4) [2.5 (3.5)]	*<0.001*
**IADL scale** [Median (Q1; Q3) [Mean (SD)]]		0 (0; 0) [0.6 (1.4)]	0 (0; 0) [0.4 (0.8)]	0 (0; 0) [0.3 (0.8)]	*0.274*	0 (0; 1) [0.8 (1.4)]	0 (0; 0) [0.4 (1.1)]	0 (0; 1) [0.6 (1.2)]	*0.066*
**Walking test** [Median (Q1; Q3)]		3.6 (3.0; 4.0)	3.5 (2.7; 4.0)	4.0 (2.6; 5.0)	*0.672*	4.4 (3.1; 6.0)	3.7 (3.2; 4.6)	3.5 (3.1; 5.0)	*0.086*
**Loneliness** [Median (Q1; Q3) [Mean (SD)]]		0.0 (0.0; 16.7) [14.4 (20.8)]	0.0 (0.0; 16.7) [12.6 (20.3)]	0.0 (0.0; 0.0) [6.2 (14.2)]	*<0.001*	0.0 (0.0; 33.3) [16.7 (22.9)]	0.0 (0.0; 16.7) [9.9 (18.6)]	0.0 (0.0; 16.7) [11.3 (19.7)]	*0.007*
**Level of social networks** [Median (Q1; Q3)]		68.9 (58.5; 74.7)	65.7 (56.1; 71.6)	61.8 (55.1; 73.5)	*0.037*	62.1 (54.8; 70.2)	68.5 (57.9; 77.6)	64.9 (55.5; 70.4)	*0.017*
**Social support** [Median (Q1; Q3)]		63.6 (54.6; 72.7)	72.7 (63.6; 81.8)	63.6 (45.5; 72.7)	*<0.001*	63.6 (54.5; 72.7)	72.7 (54.5; 81.8)	72.7 (54.5; 81.8)	*0.009*
**Trust in neighbors** (%)	Great extent^1^	29.0	30.6	29.0	*0.511*	30.8	27.1	33.7	*0.239*
	Neither great nor small	37.8	44.3	45.1		41.3	43.2	29.1	
	Small extent^2^	33.2	25.2	26.0		27.9	29.8	37.2	
**Perceived safety on the street** (%)	Completely or very safe	51.0	48.2	54.3	*0.351*	38.4	51.8	25.0	*<0.001*
	Moderately safe	30.9	40.0	31.6		28.8	39.4	38.4	
	Slightly or not safe	18.1	11.7	14.1		32.8	8.8	36.6	
**Informal social participation** [Median (Q1; Q3)]		27.2 (19.0; 41.1)	26.6 (14.3; 37.0)	27.2 (21.2; 43.9)	*0.096*	27.2 (21.2; 43.2)	26.6 (21.2; 43.2)	28.8 (17.8; 46.5)	*0.360*
**Formal social participation** [Median (Q1; Q3) [Mean (SD)]]		0.0 (0.0; 26.4) [14.0 (17.3)]	0.0 (0.0; 28.8) [14.5 (17.7)]	15.4 (0.0; 28.8) [18.2 (19.7)]	*0.225*	0.0 (0.0; 17.0) [9.8 (14.6)]	0.0 (0.0; 28.8) [12.9 (16.4)]	0.0 (0.0; 24.5) [15.5 (20.1)]	*0.005*
**Religious Service Attendance** (%)	Frequently	38.6	45.2	34.4	*0.257*	48.4	61.1	54.4	*0.005*
	Ocasionally	22.6	26.7	28.3		23.8	32.8	26.1	
	Rarely	38.8	28.1	37.3		27.8	6.1	19.5	
**COURAGE Built Environment Self-Reported Questionnaire (CBE-SR)**									
**Usability of the neighborhood environment** [Median (Q1; Q3)]		70.4 (55.1; 89.7)	56.2 (47.5; 83.9)	72.1 (59.3; 100.0)	*<0.001*	68.2 (53.8; 89.7)	59.3 (48.9; 84.9)	78.8 (56.2; 100.0)	*0.199*
**Hindrance of walkable environment** (*reversed*) [Median (Q1; Q3)]		62.2 (46.8; 77.2)	54.2 (45.0; 76.9)	64.1 (54.4; 100)	*0.056*	63.3 (44.8; 82.2)	66.0 (50.2; 83.5)	66.0 (51.2; 100.0)	*0.029*
**Easiness of use of public buildings, places and facilities** [Median (Q1; Q3)]		62.4 (57.6; 85.5)	57.6 (52.2; 85.5)	71.3 (57.6; 100)	*0.002*	57.6 (55.8; 100.0)	57.6 (48.1; 100.0)	61.2 (48.1; 100.0)	*0.851*
**Usability of the living place** [Median (Q1; Q3)]		67.7 (47.7; 88.1)	79.6 (52.1; 93.1)	73.7 (52.4; 85.4)	*0.068*	70.7 (48.8; 93.1)	76.2 (52.1; 93.1)	70.7 (48.9; 95.0)	*0.574*
**COURAGE Built Environment Outdoor Checklist (CBE-OUT)**									
**Streetscape** [Median (Q1; Q3)]		51.9 (45.7; 58.0)	51.9 (44.4; 56.8)	53.1 (48.1; 60.5)	*0.017*	53.1 (46.9; 59.3)	50.6 (45.7; 58.0)	54.3 (49.4; 61.7)	*0.011*
**Walkways** [Median (Q1; Q3)]		60.0 (53.3; 68.9)	62.2 (57.8; 66.7)	66.7 (60; 68.9)	*0.009*	64.4 (57.8; 68.9)	60.0 (57.8; 68.9)	62.2 (56.7; 68.9)	*0.807*
**Bikeways** [Median (Q1; Q3) [Mean (SD)]]		0.0 (0.0; 0.0) [9.7 (24.1)]	0.0 (0.0; 0.0) [12.8 (29.3)]	0.0 (0.0; 0.0) [9.0 (24.5)]	*0.715*	0.0 (0.0; 0.0) [9.1 (24.5)]	0.0 (0.0; 0.0) [5.6 (19.8)]	0.0 (0.0; 0.0) [8.1 (22.4)]	*0.418*
**Street crossing/intersections** [Median (Q1; Q3)]		63.2 (54.0; 67.8)	56.3 (0.0; 67.8)	65.5 (54.0; 70.1)	*<0.001*	63.2 (49.4; 67.8)	58.6 (0.0; 65.5)	65.5 (0.0; 72.4)	*0.004*
**Parking facilities** [Median (Q1; Q3)]		37.5 (25.0; 37.5)	37.5 (37.5; 37.5)	37.5 (37.5; 37.5)	*0.202*	37.5 (25.0; 37.5)	37.5 (37.5; 37.5)	37.5 (37.5; 50.0)	*0.177*
**Public facilities and features of the street** [Median (Q1; Q3)]		40.0 (20.0; 50.0)	31.9 (20.0; 40.0)	40.0 (20.0; 60.0)	*0.031*	40.0 (20.0; 50.0)	40.0 (20.0; 40.0)	40.0 (40.0; 60.0)	*0.024*
**Land-use visible along the street/road** [Median (Q1; Q3)]		61.1 (50.0; 72.2)	50.0 (38.9; 61.1)	61.1 (55.6; 72.2)	*<0.001*	61.1 (50.0; 72.2)	55.6 (44.4; 66.7)	55.6 (50.0; 66.7)	*0.007*
**Site decay/urban blight** [Median (Q1; Q3)]		81.8 (74.4; 87.0)	81.8 (74.8; 85.7)	80.6 (75.4; 85.1)	*0.752*	81.8 (74.5; 86.3)	81.6 (72.7; 86.6)	81.5 (74.5; 84.9)	*0.674*

Note: SD - Standard deviation; Q1 - first quartile; Q3 - third quartile; ^1^ - great = to a great or very great; ^2^ - small = to a small or very small; The mean and standard deviation were also provided where the differences in medians were barely noticeable.

**Table 2:** Results of the Cox proportional hazards models of associations between mobility mode and all-cause mortality across men and women aged 65+, Polish part of the COURAGE in Europe study (N = 1166). Weighted data.

Model	MEN			WOMEN		
	Slow mobility users		Fast mobility users	Slow mobility users		Fast mobility users
	Walkers	Cyclists		Walkers	Cyclists	
I	ref.	0.80 [0.52; 1.25]	0.94 [0.57; 1.56]	ref.	0.55 [0.30; 1.01]	0.74 [0.46; 1.18]
II	ref.	0.61∗ [0.39; 0.97]	0.97 [0.59; 1.56]	ref.	0.95 [0.44; 2.09]	0.80 [0.46; 1.39]
III	ref.	0.70 [0.43; 1.11]	1.03 [0.64; 1.67]	ref.	0.93 [0.42; 2.07]	0.83 [0.48; 1.43]

Note: Model I - unadjusted, Model II – adjusted for age, marital status, level of education, place of living, BMI, physical activity, grip strength, total number of diseases, visual difficulties, ADL, IADL, perceived safety on the street, Model III – additionally adjusted for loneliness, level of social networks social support, and formal participation; ∗p < 0.05.

Cox proportional hazard models were used to identify hazards of all-cause mortality. The proportional hazards assumption was assessed for all models using both statistical tests and graphical diagnostics based on scaled Schoenfeld residuals. Survival time was defined as the number of days from the interview date (ranging from April 08, 2011 to December 27, 2011) to the date of death or the date of the most recent vital status check (March 01, 2024). Variables that differed significantly across mobility modes were included as controls.

For each built environment indicator, three models were estimated: Model I – unadjusted; Model II – adjusted for age, marital status, level of education, place of living, BMI, physical activity, grip strength, total number of diseases, visual difficulties, 10.13039/100016388ADL, IADL, perceived safety on the street; Model III – further adjusted for loneliness, level of social networks social support, and formal participation (Table 3). Sensitivity analysis was conducted after exclusion of the individuals who died within the first three years after the survey (2011–2013). For two CBE-OUT indicators, Public facilities and features of the street and Site decay/urban blight, the levels of missing data were 18.9 % and 53.3 %, respectively. To deal with the missing data the linear regression models were built separately for each variable with missing data with other CBE-OUT indicators as predictor variables, and the imputed values were calculated based on the fitted regression equations. Statistical significance was set at p < 0.05. All analyses were conducted using IBM SPSS Statistics (version 29.0.2.0) and R software. Finally, mediation analyses were conducted using the *mediation* library in R to investigate whether, and to what extent, the relationship between built environment and survival time was mediated by formal, informal social participation or religious services attendance.

**Table 3:** Results of the Cox proportional hazards models of associations between build environment assessments and all-cause mortality across men and women aged 65+, Polish part of the COURAGE in Europe study (N = 1166). Weighted data.

	Model	MEN			WOMEN		
		Slow mobility users		Fast mobility users	Slow mobility users		Fast mobility users
		Walkers	Cyclists		Walkers	Cyclists	
**COURAGE Built Environment Self-Reported Questionnaire (CBE-SR)**							
Usability of the neighborhood environment	I	1.00 [0.98; 1.01]	0.99 [0.98; 1.01]	0.99 [0.98; 1.01]	0.99 [0.98; 1.01]	1.01 [0.98; 1.03]	0.99 [0.98; 1.02]
	II	0.99 [0.98; 1.01]	0.99 [0.98; 1.02]	0.97 [0.94; 1.01]	1.00 [0.99; 1.01]	1.02 [0.98; 1.07]	1.01 [0.99; 1.03]
	III	0.99 [0.98; 1.00]	0.99 [0.98; 1.02]	0.97 [0.92; 1.01]	1.00 [0.99; 1.01]	1.03 [0.96; 1.10]	0.99 [0.97; 1.02]
Hindrance of walkable environment (*reversed*)	I	0.99 [0.98; 1.01]	0.99 [0.98; 1.01]	1.00 [0.98; 1.02]	1.00 [0.99; 1.01]	0.98 [0.97; 1.01]	0.98 [0.97; 1.01]
	II	0.99 [0.98; 1.00]	0.99 [0.97; 1.02]	0.99 [0.96; 1.02]	1.00 [0.99; 1.01]	0.94∗∗∗ [0.92; 0.96]	0.99 [0.97; 1.01]
	III	0.98∗ [0.98; 0.99]	0.99 [0.97; 1.01]	0.99 [0.96; 1.03]	0.99 [0.98; 1.01]	0.95∗∗∗ [0.93; 0.97]	0.98 [0.95; 1.01]
Easiness of use of public buildings, places and facilities	I	0.99 [0.98; 1.01]	0.99 [0.97; 1.01]	0.99 [0.97; 1.01]	0.99 [0.99; 1.01]	1.01 [0.98; 1.04]	0.99 [0.98; 1.01]
	II	0.99 [0.99; 1.01]	0.99 [0.96; 1.01]	0.99 [0.95; 1.03]	0.99 [0.99; 1.01]	1.01 [0.98; 1.03]	1.01 [0.99; 1.04]
	III	0.99 [0.98; 1.00]	0.99 [0.96; 1.01]	0.99 [0.95; 1.03]	1.00 [0.99; 1.01]	1.03 [0.98; 1.07]	0.99 [0.95; 1.04]
Usability of the living place	I	1.00 [0.99; 1.01]	0.98 [0.97; 1.01]	0.99 [0.97; 1.01]	0.99 [0.99; 1.01]	0.99 [0.97; 1.02]	0.99 [0.98; 1.01]
	II	1.01 [0.99; 1.02]	0.98 [0.96; 1.01]	0.99 [0.97; 1.03]	1.01 [0.99; 1.01]	0.99 [0.977; 1.02]	1.00 [0.98; 1.02]
	III	1.01 [0.99; 1.02]	0.98 [0.96; 1.00]	1.03 [0.97; 1.10]	1.01 [0.99; 1.01]	1.01 [0.97; 1.04]	0.98 [0.95; 1.01]
**COURAGE Built Environment Outdoor Checklist (CBE-OUT)**							
Streetscape	I	0.97 [0.94; 1.01]	0.99 [0.96; 1.03]	1.01 [0.95; 1.04]	0.98 [0.96; 1.01]	0.96 [0.91; 1.01]	1.01 [0.97; 1.05]
	II	0.97 [0.94; 1.01]	1.02 [0.98; 1.07]	1.02 [0.94; 1.10]	0.98∗ [0.95; 0.99]	0.94 [0.81; 1.11]	1.01 [0.97; 1.06]
	III	0.98 [0.95; 1.01]	1.02 [0.98; 1.07]	1.03 [0.97; 1.10]	0.98∗ [0.95; 0.99]	0.88 [0.75; 1.05]	0.99 [0.94; 1.06]
Walkways	I	0.97∗ [0.95; 0.99]	0.96 [0.92; 1.01]	1.07∗∗∗ [1.03; 1.11]	1.01 [0.98; 1.03]	1.02 [0.97; 1.07]	0.98 [0.94; 1.02]
	II	0.95∗∗∗ [0.93; 0.98]	0.99 [0.95; 1.04]	1.15∗ [1.02; 1.30]	1.01 [0.99; 1.04]	1.07 [0.97; 1.19]	0.99 [0.95; 1.05]
	III	0.95∗∗∗ [0.93; 0.97]	0.99 [0.95; 1.04]	1.22∗ [1.02; 1.46]	1.01 [0.99; 1.04]	1.03 [0.94; 1.13]	0.99 [0.94; 1.05]
Bikeways	I	0.99 [0.98; 1.01]	0.99 [0.97; 1.01]	1.01 [0.99; 1.02]	0.99 [0.98; 1.01]	0.71∗∗∗ [0.69; 0.73]	1.01 [0.99; 1.03]
	II	0.99 [0.98; 1.01]	0.99 [0.96; 1.02]	1.00 [0.97; 1.04]	0.99 [0.98; 1.01]	0.69∗∗∗ [0.66; 0.72]	1.01 [0.98; 1.04]
	III	0.99 [0.99; 1.01]	0.99 [0.96; 1.02]	1.01 [0.97; 1.05]	0.99 [0.98; 1.01]	0.69∗∗∗ [0.67; 0.72]	0.99 [0.95; 1.04]
Street crossing/intersections	I	0.99 [0.98; 1.01]	0.99∗ [0.97; 0.99]	0.99 [0.98; 1.01]	0.99 [0.99; 1.01]	1.01 [0.99; 1.02]	0.99 [0.98; 1.01]
	II	0.99 [0.99; 1.01]	0.98 [0.96; 1.01]	0.98 [0.97; 1.00]	1.00 [0.99; 1.01]	0.99 [0.97; 1.01]	0.99 [0.97; 1.02]
	III	0.99 [0.99; 1.00]	0.98 [0.96; 1.00]	0.96∗∗ [0.94; 0.99]	1.00 [0.99; 1.01]	0.96 [0.92; 1.01]	0.99 [0.97; 1.02]
Parking facilities	I	1.01 [0.99; 1.02]	1.00 [0.98; 1.04]	0.98 [0.92; 1.04]	1.00 [0.99; 1.02]	1.03 [0.99; 1.07]	0.99 [0.97; 1.02]
	II	1.00 [0.99; 1.02]	1.03 [0.99; 1.07]	1.01 [0.92; 1.12]	1.01 [0.99; 1.02]	1.03 [0.94; 1.14]	1.00 [0.98; 1.03]
	III	1.00 [0.98; 1.02]	1.03 [0.99; 1.07]	0.96 [0.88; 1.06]	1.01 [0.99; 1.02]	1.05 [0.94; 1.16]	0.99 [0.96; 1.03]
Public facilities and features of the street	I	0.99 [0.98; 1.01]	0.99 [0.96; 1.01]	1.03∗ [1.01; 1.05]	0.99 [0.98; 1.01]	0.98 [0.96; 1.02]	0.98 [0.95; 1.01]
	II	0.99 [0.98; 1.01]	1.01 [0.98; 1.05]	1.08 [0.98; 1.08]	0.99 [0.98; 1.00]	1.02 [0.96; 1.07]	0.98 [0.95; 1.01]
	III	0.99 [0.98; 1.01]	1.01 [0.98; 1.05]	1.04 [0.98; 1.11]	0.99 [0.98; 1.01]	1.02 [0.92; 1.14]	0.96∗∗∗ [0.93; 0.98]
Land-use visible along the street/road	I	0.98∗ [0.97; 0.99]	0.99 [0.97; 1.02]	1.01 [0.98; 1.05]	0.99 [0.98; 1.01]	0.99 [0.94; 1.04]	0.99 [0.97; 1.02]
	II	0.98 [0.97; 1.01]	1.01 [0.96; 1.05]	1.03 [0.97; 1.09]	0.99 [0.97; 1.01]	0.98 [0.92; 1.04]	0.99 [0.93; 1.05]
	III	0.98 [0.97; 1.01]	1.01 [0.96; 1.05]	1.01 [0.93; 1.10]	0.99 [0.97; 1.01]	0.83 [0.66; 1.05]	0.96 [0.90; 1.03]
Site decay/urban blight	I	1.00 [0.98; 1.01]	1.03 [0.97; 1.09]	1.01 [0.97; 1.05]	0.99 [0.98; 1.01]	1.07 [0.99; 1.14]	0.99 [0.96; 1.04]
	II	0.99 [0.98; 1.02]	1.01 [0.94; 1.09]	0.98 [0.91; 1.05]	0.99 [0.98; 1.02]	1.15 [0.96; 1.37]	0.98 [0.94; 1.02]
	III	0.99 [0.98; 1.02]	1.01 [0.94; 1.09]	0.97 [0.86; 1.09]	0.99 [0.97; 1.02]	1.28 [0.87; 1.91]	0.99 [0.92; 1.06]

Note: Model I - unadjusted, Model II – adjusted for age, marital status, level of education, place of living, BMI, physical activity, grip strength, total number of diseases, visual difficulties, ADL, IADL, perceived safety on the street; Model III – additionally adjusted for loneliness, level of social networks social support, and formal participation; ∗p < 0.05; ∗∗p < 0.01; ∗∗∗p < 0.001.

An accelerated failure time (AFT) model was employed as the outcome model, as the proportional hazards model lacks a clear causal interpretation of the effect when applied to non-rare outcomes (VanderWeele, 2011).

## 5 Results

Among those who move autonomously, walking was the leading mode of mobility in local neighborhood, and it was reported by 90.7 % of women and 82.6 % of men. Cycling was reported by 9.5 % of women and 20.4 % of men. The third most frequent mode of mobility for men was private motor vehicle use (12.8 % compared to 6.2 % for women), while for women it was public transport (8.2 % vs. 8.5 % in men). Walking with dog concerned 3.4 % of women and 8.3 % of men, and 2.9 % of women and 1.1 % of men used personal mobility aid(s).

Regarding movement habits**,** 77.2 % of women and 60.7 % men usually only walk (slow mobility users – walkers), 20.4 % of women and 9.5 % men usually cycle (slow mobility users – cyclists) around their neighborhood, and 13.3 % of women and 18.9 % men are fast mode transportation users (Table 1).

### 5.1 Comparison of the individual characteristics in men and women across the mobility modes

#### 5.1.1 Slow mobility users - walkers

The results showed that the group of men who primarily walked were more likely to live in urban areas (compared to cyclists), had the lowest BMI, reported mild or moderate visual difficulties, reported the highest level of social networks. They also assessed the quality of walkways the worst (Table 1).

Women in the walking group within slow mobility users were more likely to be older (compared to cyclists), less likely to be married, more likely to live in urban areas, had low physical activity, low grip strength, more inabilities (ADL), and chronic diseases, as well as lower levels of social networks and support, lower levels of formal participation, and a higher level of loneliness. They were more likely to live in places where the types of buildings and open-to-public green spaces (land-use visible along the street) were more conducive to participation in the local community (Table 1).

#### 5.1.2 Slow mobility users - cyclists

Men aged 65+ who reported cycling as one of the two most frequent modes of mobility in their neighborhood were more likely to have a primary education and live in rural areas (compared to the walkers and fast mobility groups). They had a lower BMI compared to fast mobility group and reported the highest level of social support. This group of men assessed the usability of the neighborhood environment, public buildings, places and facilities the worst compared to walkers and fast mobility groups (Table 1).

Women were more likely to be younger, married, and most of them lived in rural areas, compared to walkers and fast mobility users. They were more frequently involved in high physical activity, had normal grip strength, and demonstrated better functional ability in terms of ADL, as well as a lower number of diseases. Women who reported cycling most often indicated that they felt very safe on the street. They reported the highest level of social networks and the lowest level of loneliness (Table 1).

In areas were men and women cycled, the objective assessment of built environment showed that street crossing and land-use visible along the street were less conducive to participation by ageing members of the local community. Public facilities and street features were also less accessible, and the streetscape was significantly less useable and comfortable (Table 1.).

#### 5.1.3 Fast mobility users

Fast mobility men were more likely to have completed high school or university education. They were more likely to live in urban areas and more frequently had a higher BMI. This group of men reported the lowest level of loneliness, but also the lowest level of social networks. They rated the usability of the neighborhood and public building accessibility the highest (Table 1).

Fast mobility women were also more likely to live in urban areas. They were more likely to have normal grip strength compared to the walkers, but less likely than the cyclists. They reported higher level of social support compared to the walkers and higher level formal social participation relative to both slow mobility groups. The level of social network was higher than that of the walkers, but lower than that of the cyclists. This group of women perceived the fewest visible hindrances to walkability (Table 1).

The objective assessment of the built environment for fast mobile women showed that their streetscape had the highest number of facilities. In these areas, street crossings, public facilities, and street features had a more positive impact on neighborhood accessibility for healthy aging compared to the environments where walkers and cyclists lived. The environment were men lived had more facilities in terms of streetscape, walkways, street crossings, public amenities, features of the street and visible land use (Table 1).

#### 5.1.4 The relationship between mobility mode and all-cause mortality

In men, those who move by cycle had significantly lower risk of mortality when compared to the walkers (HR = 0.61) in model controlled for sociodemographic, health and functional characteristics, but not after controlling for other psychosocial variables. There were no statistically significant differences in the risk of death between groups according to mobility mode in women (Table 2). The sensitivity analyses did not confirmed the results (Supplementary Table S7).

#### 5.1.5 The relationship between self-reported features of built environment (CBE-SR) and all-cause mortality in men and women across mobility modes

Reduced hindrance of walkable environment decreased the risk of mortality in fully adjusted model for walking group of men (HR = 0.98) and in women who reported cycling as one of two the most frequent mode of mobility (HR = 0.95 for model III) (Table 3). The results for men and women (for model II) were also significant after exclusion of people who died during the first 3 years of follow-up (see Supplementary Table S6).

#### 5.1.6 The relationship between objective features of built environment (CBE-OUT) and all-cause mortality in men and women across mobility modes

In the walking group of men better-quality walkways decrease the risk of mortality in unadjusted model and after controlling for considered covariates (HR = 0.95). Sensitivity analysis confirmed the result. Moreover, land use visible along the street/road facilitating modes of mobility for older people decreased the risk of death, but only in unadjusted model, and not after exclusion of participants who died during the initial three years. In women, the significant protective role of higher number of facilities on visible streetscape (more age-friendly streetscape) was observed for the walkers group (HR = 0.98) in adjusted models, also in sensitivity models. (Table 3 and Supplementary Table S6).

For men who cycle in their local area, higher quality of street crossing was found as protective factor for the risk of death but only in unadjusted model. In women, higher quality of bikeways significantly decreased the risk of death (HR = 0.69 in model III). Sensitivity analysis supported the results (Table 3 and Supplementary Table S6).

In men from the fast mobility group, higher quality of street crossing was identified as a protective factor (HR = 0.96 in model III). The finding was confirmed by sensitivity analysis. Higher scores on the scale ‘public facilities and features of the street” was associated with lower risk of death among women who use motor vehicles or public transport to move in their neighborhood, in fully-adjusted model (HR = 0.96 in model III). In men, this factor increased the risk of death but only in unadjusted model. The same was observed after exclusion individuals who died during the first 3 years of follow-up (Table 3 and Supplementary Table S6).

In men better-quality walkways significantly increased the risk of death for fast mobility group (HR = 1.22 in model III), but sensitivity analysis did not confirm the results in adjusted models (Table 3 and Supplementary Table S6). After exclusion of the people who died during the first 3 years of follow-up higher quality of bikeways was found as risk factor for higher mortality in men (HR = 1.06 in model III) (Supplementary Table S6).

#### 5.1.7 Social participation as mediator of the relationship between BE characteristics and mortality

The results of the mediation analyses for different modes of mobility in women and men are presented in Supplementary Tables S3, S4 and S5. These analyses identified that both formal and informal social participation mediate the relationship between public facilities and features of the street and survival time in women (Table S3 and S4). Informal social participation and attendance at religious activities significantly mediate the relationship between bikeways and survival time in the walking group of women. However, the effect was reversed, showing reduced survival time (Table S4 and S5). In men, informal social participation mediates the association between hindrance of walkable environment and survival time (Table S4). All results were not significant in adjusted models.

## 6 Discussion

This study examined the relationship between mobility modes and all-cause mortality in men and women over a 12.5-year period. Women who reported at baseline that they usually move around autonomously in their neighborhood by cycling had lower mortality than those who did so by walking, using public transportation, or driving a private motor vehicle. In men, the results were not significant. Second, we examined which aspects of the subjective and objective assessment of the built environment were related to risk of death. A subjective evaluation of a walkable environment with fewer hindrances decreased the risk of mortality for the walking group of men and women who reported cycling, even when models were controlled for age, marital status, level of education, place of living, BMI, physical activity, grip strength, total number of diseases, visual difficulties, ADL, IADL, perceived safety on the street, loneliness, level of social networks social support, and formal participation. Among the objective features of the built environment, the higher quality of walkways for men and a higher number of facilities on the visible streetscape for women were found to be protective factors for the risk of death. For cyclists, the higher quality of bikeways decreased the risk of death among women. In men, only the higher quality of street crossings was found to be protective factor, but was not significant in the adjusted models. Among those who also use public transport or cars to move autonomously in the local area, higher number of public facilities and street features were associated with a lower risk of death in women. In men, however, this factor was associated with an increased risk of death, but only in the unadjusted model. In men, the higher quality of street crossings was found to be a protective factor. Surprisingly, better-quality walkways were related to a higher risk of death in the fast mode of transportation group of men.

In our study, we found that walking was the most frequently indicated type of mobility around one's neighborhood. Women reported walking more frequently than men. According to Rantakokko et al. (2015) walking, driving and using public transport were the main forms of mobility for older adults in their local neighborhood. This is in line with other studies (Tournier et al., 2016), which were also conducted at that time in Poland. A 2007 study among people aged 60–69 found that women had higher levels of physical activity than men, with walking being the preferred form of activity (86.8 % of women and 40 % of men) (Pocztarska-Dec & Bergier, 2012). This may be related to a greater willingness among women to participate in active aging promoting programs (e.g., Nordic walking, outdoor gyms, etc.). At the same time, men in this age group are more likely to possess a driving license, and therefore are more likely to be able to drive a car.

The study also highlighted that cycling was definitely the most common mode of transportation in rural areas in Poland, where the availability of public transport was less common than in urban places. According to POLSenior 1 (Wizner et al., 2012), conducted among a representative sample of older Polish adults, a total of 64.5 % of respondents (57.3 % of women and 71.3 % of men) used public transport or taxis without assistance, while 10.8 % (12.3 % of women and 9.4 % of men) used them with assistance.

Polish data, based on research conducted in 2000–2001 as part of the WHO's CINDI programme, showed that in urban environments the percentage of older adults with low physical activity was 60 % for men and 70 % for women. In the WOBASZ study (conducted in 2002–2004), 55 % of women and 49 % of men aged 20–74 were characterized by low levels of leisure-time physical activity (Drygas et al., 2005). A study of 420 randomly selected households in the city center of Lodz found that older people often walked to do their daily shopping (40–50 % of respondents aged 60), and they walked to church (an average distance of up to 800 m) (Borowska-Stefańska & Wiśniewski, 2019). Older people positively evaluated well-located bus stops (300 m) (Borowska-Stefańska & Wiśniewski, 2019).

The results of our study showed that the risk of death in older adults was lower among men who cycle, compared to the walking group, but only in the non-fully adjusted model and not in the sensitivity model. This is in line with other studies, which showed that both recreational and commuter cycling up to 60 min per week reduce the risk of all-cause mortality compared to those who never cycle (Østergaard et al., 2018). Cycling in this context might be considered a form of physical activity, but some scientists also emphasize the role of well-being. Previous studies have shown that cycling is associated with better well-being compared to bus and car commuters (Smith, 2017). Cycling is considered more satisfactory than other modes of transportation due to the following reasons: a high degree of commuting time control, enjoyable sensory stimulation from combining muscular effort with sensory input from the landscape, the effects of moderate-intensity exercise, and greater opportunities for social interaction (Wild & Woodward, 2019).

The result of our study also showed that, for women, a more age-friendly pedestrian streetscape was a significant protective factor against the risk of death in the walking group. In particular, among the group of walking men, the higher quality of walkways and the subjective assessment of a hindering of walkable environment were important determinants of mortality. A previous study conducted on 727 community-dwelling people aged 75–81 indicated that barriers in the outdoor environment, such as terrain, traffic and distances were related to fear of moving outdoors and reduced physical activity, which also influenced quality of life (Rantakokko et al., 2010). Fear of moving outdoors also increased the risk of developing self-reported difficulties in walking between 0.5 km and 2 km (Rantakokko et al., 2010). Numerous studies show that limitations in mobility increase the risk of mortality in older age (do Nascimento et al., 2022; Abizanda et al., 2013; Otsuka et al., 2021). Polku et al. (2015) showed that poorer life space mobility was associated with higher depressive symptoms. The associations were partially mediated by walking difficulty, health, and sense of autonomy from participation in outdoor activities. Environmental conditions affect the outdoor mobility of older adults, either by facilitating or restricting participation in activities outside the home (Rantakokko et al., 2015). Other environmental barriers to outdoor mobility, as subjectively rated by older adults, include poor transportation, discontinuous or uneven sidewalks, curbs, noise, heavy traffic, inadequate lighting, lack of resting places, sloping terrain, long distance to services, weather conditions and poor road conditions (Rantakokko et al., 2015). The importance of the quality of urban environments, whether supportive of older people's mobility or indicative of the existence of multiple barriers is emphasised in ecological models. Lawton and Nahemow (1973), developed a model of adaptation in older age to examine an individual's ability to interact successfully with the demands of the environment. Patla and Shumway-Cook (1999) described a mobility continuum and showed a positive relationship between independent walking endurance and accessibility to the community.

In the present study, the presence of continuous bikeways was found to decrease the risk of death among women who cycle. This aligns, with studies indicating that alterations to the road surface are a predictor of cyclist mortality (Molina-Soberanes et al., 2019) and purpose-built bicycle only facilities reduce the risk of crashes and injuries (Reynolds et al., 2009).

Our study also showed that higher number of public facilities and street features are important protective factors of all-cause mortality among women who use private or public motor vehicles. This group of women had better health and functional status compared to the walking group and was more likely to take advantage of these facilities.

Surprisingly, better-quality walkways and bikeways (after exclusion of the first three years of follow-up) were associated with a higher risk of death in the fast mode of transportation group of men. It might be link to the fact that infrastructure designed for slow mobility users requires greater attention from pedestrians, cyclists and other road users, which may be more challenging for older adults. Bicycle lanes as sources of conflicts between different road users were pointed out by Schepers et al. (2015).

Finally, significant indirect effects of formal and informal social participation were observed in the relationship between public facilities and features of the street and all-cause mortality, in the women's walking group. In men in the fast mobility users group, informal social participation significantly mediated the association between hindrance of walkable environment and survival time. Our results were not significant after controlling for sociodemographic, health and functional characteristics. The results support the previous finding that social participation mediate the association between the acceptance of the leisure environment and the landscape environment and health (Zheng et al., 2022). Nonetheless, the other pathways are possible e.g. through physical activity (Wan et al., 2022; Zheng et al., 2022), air pollutants (Feng et al., 2023; Wan et al., 2022), subjective well-being (He et al., 2023), lessening social isolation (Wan et al., 2022) or relieving depression (Wan et al., 2022).

Statistically significant indirect effects were also observed in the relationships between informal social participation and bikeways, as well as between attendance at religious activities and bikeways, in relation to survival time in the walking group of women. However, the results indicated that through these pathways, survival time was reduced. They were only significant in the unadjusted models. This may suggest that the presence of bicycle lanes was treated as a possible barrier for older walkers, especially for those with mobility limitations. The findings could be related to possible conflicts between various road users (Schepers et al., 2015).

### 6.1 Strength and limitation of the study

Strength of the study include a relatively large and randomly selected sample of people aged 65+ from Poland. Subjective and objective detailed measures of built environment from an age–friendly perspective were assessed. A wide group of covariates was considered, including both self-reported data and anthropometric measures. The validity of all-cause mortality as the outcome is also considered to be very high in Poland.

The findings of this study should be considered in light of some limitations. Considered variables were recorded at baseline (2011) and might likely have changed over the 12.5 years of follow-up. Second, we did not have direct measurements of air, heat and noise pollution, which might have an impact on mortality in a variety of ways. Third, since the baseline study was cross-sectional it was not possible to verify if the type of mobility was a result or a cause of better heath and functional status at that time.

## 7 Conclusion

The observed patterns of relationships between built environment indicators and all-cause mortality differed for men and women, as well as for modes of mobility. Subjective characteristics of the built environment such as reduced hindrance of walkable environment decreased the risk of death of men who mainly walk, and women who cycle. Objective characteristics of the built environment, such as streetscapes (in women who mainly walk in their neighborhood) and walkways (in men who mainly walk in their neighborhood) for walkers, and bikeways (in women who cycle) were found to be significant protective factors against mortality, emphasizing the importance of planning and organizing the built environment from an age–friendly perspective. The study showed that among various built environment characteristics, improving the walkable environment may significantly reduce mortality in older people. At the same time, the study revealed higher all-cause mortality among the fast mode group of men when continuous walkways or bikeways were present, highlighting the need for a holistic approach to transport infrastructure planning.

## CRediT authorship contribution statement

**Katarzyna Zawisza:** Writing – review & editing, Writing – original draft, Validation, Project administration, Methodology, Investigation, Funding acquisition, Data curation, Conceptualization. **Michalina Gajdzica:** Writing – original draft, Visualization, Validation, Formal analysis, Data curation. **Alberto Raggi:** Writing – review & editing. **Beata Tobiasz-Adamczyk:** Writing – review & editing, Writing – original draft, Supervision, Investigation, Funding acquisition, Conceptualization.

## Ethical statement

The Jagiellonian University Bioethics Committee approved the study protocol [no. 122.6120.262015, 118.6120.125.2023]. Written informed consent to participate in the study was obtained from each participant.

## Declaration of generative AI in scientific writing

Not used. Nothing to declare.

## Funding

The research leading to these results has received funding from the European Community's Seventh Framework Programme (FP7/2007–2013) under grant agreement number 223071 (COURAGE in Europe), from the Polish Ministry for Science and Higher Education grant for an international co-financed project (number 1277/7PR/UE/2009/7, 2009–2012); further study related to mortality was founded by Jagiellonian University Medical College, Poland (the Jagiellonian University Medical College grant (N41/DBS/001031)).

## Declaration of competing interest

The authors declare that they have no known competing financial interests or personal relationships that could have appeared to influence the work reported in this paper.

## Data Availability

Data will be made available on request.

## References

[bib1] Abizanda P., Romero L., Sánchez-Jurado P.M., Martínez-Reig M., Gómez-Arnedo L., Alfonso S.A. (2013). Frailty and mortality, disability and mobility loss in a Spanish cohort of older adults: the FRADEA study. Maturitas.

[bib2] Barnett D.W., Barnett A., Nathan A., Van Cauwenberg J., Cerin E., Council on Environment and Physical Activity (CEPA)–Older Adults working group (2017). Built environmental correlates of older adults' total physical activity and walking: A systematic review and meta-analysis. International Journal of Behavioral Nutrition and Physical Activity.

[bib3] Borowska-Stefańska M., Wiśniewski S. (2019).

[bib4] Czarnecki P., Podgórska-Bednarz J., Perenc L. (2020). Forms of physical activity of the elderly. European Journal of Clinical and Experimental Medicine.

[bib5] Dalgard O., Trent D., Reed C. (1996).

[bib6] do Nascimento C.F., Lay A.A.R., Duarte Y.A.O., Chiavegatto Filho A.D.P. (2022). Functional mobility and 10-year all-cause and cause-specific mortality in older people from São Paulo, Brazil. Brazilian Journal of Physical Therapy.

[bib7] Drygas W., Kwaśniewska M., Szcześniewska D., Kozakiewicz K., Głuszek J., Wiercińska E., Wyrzykowski B., Kurjata P. (2005). Ocena poziomu aktywności fizycznej dorosłej populacji polski. Wyniki programu WOBASZ. Kardiologia Polska.

[bib8] EU (2021). THE NEW EUROPEAN urban mobility framework. https://ec.europa.eu/commission/presscorner/detail/en/fs_21_6781.

[bib9] Feng C., Yu B., Fei T., Jia P., Dou Q., Yang S. (2023). Association between residential greenness and all-cause mortality and the joint mediation effect of air pollutants among old people with disability: a prospective cohort study. Science of the Total Environment.

[bib10] Freiberger E., Sieber C.C., Kob R. (2020). Mobility in older community-dwelling persons: A narrative review. Frontiers in Physiology.

[bib11] Groessl E.J., Kaplan R.M., Rejeski W.J., Katula J.A., Glynn N.W., King A.C., Anton S.D., Walkup M., Lu C.-J., Reid K., Spring B., Pahor M. (2019). Physical activity and performance impact long-term quality of life in older adults at risk for major mobility disability. American Journal of Preventive Medicine.

[bib12] He Q., Liu L., Zhang H., Chen R., Dong G., Yan L.L., Zeng Y., Kim Y., Ji J.S. (2023). Environmental greenspace, subjective well-being, and all-cause mortality in elderly Chinese: Association and mediation study in a prospective cohort. Environmental Research.

[bib13] Hughes M.E., Waite L.J., Hawkley L.C., Cacioppo J.T. (2004). A short scale for measuring loneliness in large surveys: Results from two population-based studies. Research on Aging.

[bib14] Joseph A., Zimring C., Harris-Kojetin L., Kiefer K. (2006). Presence and visibility of outdoor and indoor physical activity features and participation in physical activity among older adults in retirement communities. Journal of Housing for the Elderly.

[bib15] Kawachi I., Berkman L.F., Berkman L.F., Kawachi I. (2000). Social epidemiology.

[bib16] Kennedy R.E., Sawyer P., Williams C.P., Lo A.X., Ritchie C.S., Roth D.L., Allman R.M., Brown C.J. (2017). Life-space mobility change predicts 6-month mortality. Journal of the American Geriatrics Society.

[bib17] Khokhar S.R., Stern Y., Bell K., Anderson K., Noe E., Mayeux R., Albert S.M. (2001). Persistent mobility deficit in the absence of deficits in activities of daily living: A risk factor for mortality. Journal of the American Geriatrics Society.

[bib18] Kramer A.F., Erickson K.I. (2007). Effects of physical activity on cognition, well-being, and brain: Human interventions. Alzheimer’s & Dementia.

[bib19] Lawton M.P., Nahemow L., Eisdorfer C., Lawton M.P. (1973). The psychology of adult development and aging.

[bib20] Lee I.M., Buchner D.M. (2008). The importance of walking to public health. Medicine & Science in Sports & Exercise.

[bib21] Leonardi M., Chatterji S., Koskinen S., Ayuso-Mateos J.L., Haro J.M., Frisoni G., Frattura L., Martinuzzi A., Tobiasz-Adamczyk B., Gmurek M., Serrano R., Finocchiaro C., COURAGE in Europe Project's Consortium (2014). Determinants of health and disability in ageing population: The COURAGE in Europe project (collaborative research on ageing in Europe). Clinical Psychology & Psychotherapy.

[bib22] Lin J., Leung J., Yu B., Woo J., Kwok T., Lau K.K.L. (2021). Socioeconomic status as an effect modifier of the association between built environment and mortality in elderly Hong Kong Chinese: A latent profile analysis. Environmental Research.

[bib23] Lindström M., Rosvall M. (2019). Two theoretical strands of social capital, and total, cardiovascular, cancer and other mortality: A population-based prospective cohort study. SSM - Population Health.

[bib24] Liu Y., Dijst M., Faber J., Geertman S., Cui C. (2017). Healthy urban living: Residential environment and health of older adults in shanghai. Health & Place.

[bib25] Mackey D.C., Cauley J.A., Barrett-Connor E., Schousboe J.T., Cawthon P.M., Cummings S.R., Osteoporotic Fractures in Men Research Group (2014). Life-space mobility and mortality in older men: a prospective cohort study. Journal of the American Geriatrics Society.

[bib26] Molina-Soberanes D., Martínez-Ruiz V., Lardelli-Claret P., Pulido-Manzanero J., Martín-delosReyes L.M., Moreno-Roldán E., Jiménez-Mejías E. (2019). Individual and environmental factors associated with death of cyclists involved in road crashes in Spain: a cohort study. BMJ Open.

[bib27] Mori Y., Tsuji T., Watanabe R., Hanazato M., Chen Y.R., Kondo K. (2023). Built environments and frailty in older adults: The JAGES longitudinal study using mediation analysis. Journal of the American Medical Directors Association.

[bib28] Newman A.B., Simonsick E.M., Naydeck B.L., Boudreau R.M., Kritchevsky S.B., Nevitt M.C., Pahor M., Satterfield S., Brach J.S., Studenski S.A., Harris T.B. (2006). Association of long-distance corridor walk performance with mortality, cardiovascular disease, mobility limitation, and disability. JAMA.

[bib29] Østergaard L., Jensen M.K., Overvad K., Tjønneland A., Grøntved A. (2018). Associations between changes in cycling and all-cause mortality risk. American Journal of Preventive Medicine.

[bib30] Otsuka H., Kobayashi H., Suzuki K., Hayashi Y., Ikeda J., Kushimoto M., Hara M., Abe M., Kato K., Soma M. (2021). Mobility performance impacts mortality risk in community-dwelling healthy older adults in Japan: A prospective observational study. Aging Clinical and Experimental Research.

[bib31] Patla A.E., Shumway-Cook A. (1999). Dimensions of mobility: Defining the complexity and difficulty associated with community mobility. Journal of Aging and Physical Activity.

[bib32] Pocztarska-Dec A., Bergier J. (2012). Aktywność ruchowa ludzi starszych w świetle dotychczasowych badań. Człowiek i Zdrowie.

[bib33] Polku H., Mikkola T.M., Portegijs E., Rantakokko M., Kokko K., Kauppinen M., Rantanen T., Viljanen A. (2015). Life-space mobility and dimensions of depressive symptoms among community-dwelling older adults. Aging & Mental Health.

[bib34] Quintas R., Raggi A., Bucciarelli P., Franco M.G., Andreotti A., Caballero F.F., Olaya B., Chatterji S., Galas A., Meriläinen-Porras S., Frisoni G., Russo E., Minicuci N., Power M., Leonardi M. (2014). The COURAGE built environment outdoor checklist: An objective built environment instrument to investigate the impact of the environment on health and disability. Clinical Psychology & Psychotherapy.

[bib35] Raggi A., Corso B., De Torres L., Quintas R., Chatterji S., Sainio P., Martinuzzi A., Zawisza K., Haro J.M., Minicuci N., Leonardi M. (2018). Determinants of mobility in populations of older adults: Results from a cross-sectional study in Finland, Poland and Spain. Maturitas.

[bib36] Raggi A., Quintas R., Bucciarelli P., Franco M.G., Andreotti A., Miret M., Zawisza K., Olaya B., Chatterji S., Sainio P., Frisoni G.B., Martinuzzi A., Minicuci N., Power M., Leonardi M. (2014). Validation of the COURAGE built environment self-reported questionnaire. Clinical Psychology & Psychotherapy.

[bib37] Rantakokko M., Iwarsson S., Kauppinen M., Leinonen R., Heikkinen E., Rantanen T. (2010). Quality of life and barriers in the urban outdoor environment in old age. Journal of the American Geriatrics Society.

[bib38] Rantakokko M., Iwarsson S., Portegijs E., Viljanen A., Rantanen T. (2015). Associations between environmental characteristics and life-space mobility in community-dwelling older people. Journal of Aging and Health.

[bib39] Rantanen T., Portegijs E., Viljanen A., Eronen J., Saajanaho M., Tsai L.T., Kauppinen M., Palonen E.M., Sipilä S., Iwarsson S., Rantakokko M. (2012). Individual and environmental factors underlying life space of older people - Study protocol and design of a cohort study on life-space mobility in old age (LISPE). BMC Public Health.

[bib40] Reynolds C.C.O., Harris M.A., Teschke K., Cripton P.A., Winters M. (2009). The impact of transportation infrastructure on bicycling injuries and crashes: A review of the literature. Environmental Health.

[bib41] Rojas-Rueda D., Nieuwenhuijsen M.J., Gascon M., Perez-Leon D., Mudu P. (2019). Green spaces and mortality: A systematic review and meta-analysis of cohort studies. The Lancet Planetary Health.

[bib42] Scharlach A.E., Lehning A.J. (2013). Ageing-friendly communities and social inclusion in the United States of America. Ageing and Society.

[bib43] Schepers P., Fishman E., Beelen R., Heinen E., Wijnen W., Parkin J. (2015). The mortality impact of bicycle paths and lanes related to physical activity, air pollution exposure and road safety. Journal of Transport & Health.

[bib44] Seinsche J., Jansen C.P., Roth S., Zijlstra W., Hinrichs T., Giannouli E. (2023). Multidimensional interventions to increase life-space mobility in older adults ranging from nursing home residents to community-dwelling: A systematic scoping review. BMC Geriatrics.

[bib45] Smith O. (2017). Commute well-being differences by mode: Evidence from Portland, Oregon, USA. Journal of Transport & Health.

[bib46] Sprague B.N., Zhu X., Ehrenkranz R.C., Tian Q., Gmelin T.A., Glynn N.W., Rosso A.L., Rosano C. (2021). Declining energy predicts incident mobility disability and mortality risk in healthy older adults. Journal of the American Geriatrics Society.

[bib47] Takano T., Nakamura K., Watanabe M. (2002). Urban residential environments and senior citizens' longevity in megacity areas: The importance of walkable green spaces. Journal of Epidemiology & Community Health.

[bib48] Tournier I., Dommes A., Cavallo V. (2016). Review of safety and mobility issues among older pedestrians. Accident Analysis & Prevention.

[bib49] VanderWeele T.J. (2011). Causal mediation analysis with survival data. Epidemiology.

[bib50] Wan S., Rojas-Rueda D., Pretty J., Roscoe C., James P., Ji J.S. (2022). Greenspace and mortality in the U.K. biobank: Longitudinal cohort analysis of socio-economic, environmental, and biomarker pathways. SSM - Population Health.

[bib51] Webber S.C., Porter M.M., Menec V.H. (2010). Mobility in older adults: A comprehensive framework. The Gerontologist.

[bib52] Wild K., Woodward A. (2019). Why are cyclists the happiest commuters? Health, pleasure and the e-bike. Journal of Transport & Health.

[bib53] Wizner B., Skalska A., Klich-Rączka A., Piotrowicz K., Grodzicki T., Mossakowska M., Więcek A., Błędowski P. (2012). POLSENIOR 1.

[bib54] Zawisza K., Galas A., Tobiasz-Adamczyk B., Chatterji S., Haro J.M., Miret M., Koskinen S., Power M., Leonardi M. (2014). The validity of the instrument to evaluate social network in the ageing population: The collaborative research on ageing in Europe social network index. Clinical Psychology & Psychotherapy.

[bib55] Zawisza K., Sekuła P., Gajdzica M., Tobiasz-Adamczyk B. (2024). Social capital and all-cause mortality before and during the COVID-19 pandemic among middle-aged and older people: Prospective cohort study in Poland. Social Science & Medicine.

[bib56] Zheng Z., Liu W., Lu Y., Sun N., Chu Y., Chen H. (2022). The influence mechanism of community-built environment on the health of older adults: From the perspective of low-income groups. BMC Geriatrics.

