# Supplementary material for: The impact of slow mobility and built environment characteristics on 12.5-year all-cause mortality among older women and men: A prospective cohort study from Poland
Source: SSM Popul Health. 2025 Jul 11;31:101841. doi: 10.1016/j.ssmph.2025.101841 (PMC12281007; doi:10.1016/j.ssmph.2025.101841)
Supplement: Multimedia component 1 [file mmc1.docx]

**The Impact of Slow Mobility and Built Environment Characteristics on 12.5-Year All-Cause Mortality Among Older Women and Men: A Prospective Cohort Study from Poland.**

Supplementary materials

Table of contents

[Figure S1. Model of the study. 2](#_Toc202956096)

[Figure S2. Flow diagram of the study population, Polish part of the COURAGE in Europe study. 3](#_Toc202956097)

[Table S1. Baseline characteristics by mobility mode among men and women aged 65+, Polish part of the COURAGE in Europe study (N = 1313), 2011. Unweighted data. Non-ambulatory group was not included in the main manuscript. 4](#_Toc202956098)

[Table S2. Baseline characteristics by mobility mode among men and women aged 65+, Polish part of the COURAGE in Europe study (N = 1313), 2011. Weighted data. Non-ambulatory group was not included in the main manuscript. 8](#_Toc202956099)

[Figure S3. Assumed causal model. Diagram of the assumed associations between built environment on survival time through the potential social participation mediators. 12](#_Toc202956100)

[Table S3. Results of mediation analysis: The effect of built environment on survival time through formal social participation (N = 1166). Weighted data. 13](#_Toc202956101)

[Table S4. Results of mediation analysis: The effect of built environment on survival time through informal social participation (N = 1166). Weighted data. 15](#_Toc202956102)

[Table S5. Results of mediation analysis: The effect of built environment on survival time through religious services attendance (N = 1166). Weighted data. 17](#_Toc202956103)

[Table S6. Sensitivity analysis. Results of the Cox proportional hazards models of associations between build environment assessments and all-cause mortality across men and women aged 65+, Polish part of the COURAGE in Europe study, after excluding individuals who died between 2011 and 2013 (N = 1166). Weighted data. 18](#_Toc202956104)

[Table S7. Sensitivity analysis. Results of the Cox proportional hazards models of associations between mobility mode and all-cause mortality across men and women aged 65+, Polish part of the COURAGE in Europe study, after excluding individuals who died between 2011 and 2013 (N = 1166). Weighted data. 21](#_Toc202956105)

# Figure S1. Model of the study.

Mobility mode:

**Slow** (only walking, walking with dog, walking with aids)

**Fast** (mainly private motor-vehicles and public transport)

**Cycling** (mainly bicycles)

Build environment indicators:

*COURAGE Built Environment Self-Reported Questionnaire* (usability of the neighborhood
environment, hindrance of walkable environment, easiness of use of public buildings, places and facilities, usability of the living place)

*COURAGE Built Environment Outdoor Checklist* (streetscape, walkways, bikeways, street crossing/intersections, parking facilities, public facilities and features of the street, land-use visible along the street/road, site decay/urban blight)

Covariates:

Demographic and socioeconomic characteristics, health-related behaviors, health and functioning status, psychosocial factors

**MEN**

4427.9 person years

Non-ambulatory respondents were excluded.

**Men** (N=473; 266 deaths (56.2%))

**Women** (N=693, 311 deaths (44.9%))

**All-cause mortality** (from 08.04.2011-27.12.2011 to 01.03.2024)

**Men** (N=510; 295 deaths)

**Women** (N=803; 409 deaths)

**WOMEN**

7648.5 person years

**MEN** without non-ambulatory group

4204.9 person years

**WOMEN** without non-ambulatory group

6973.8 person years

# Figure S2. Flow diagram of the study population, Polish part of the COURAGE in Europe study.

Excluded non-ambulatory group

(n=147 (11.2%))

Analyzed variables with any missing values:

BMI (n=54), walking test (n=408), score social support (n=7), usability of the neighborhood
environment (n=11), hindrance of walkable environment (n=116), easiness of use of public buildings, places and facilities (n=168), usability of the living place (n=97), streetscape (n=43), walkways (n=41), bikeways (n=41), street crossing/intersections (n=41), parking facilities (n=49), public facilities and features of the street (n=57), land-use visible along the street/road (n=48), site decay/urban blight (n=66)

Total COURAGE sample in Poland

(n=4071)

Respondents aged 65 years and older

(n=1412 (34.7%))

Dataset after proxy exclusion

(n=1313 (92.9 %))

(men: n=510 (38.8%), women: n=803 (61.2%))

Respondents aged 18 to 64

(n=2652 (65.1%))

Individuals excluded due to inconsistences between date of death and date of the interview

(n=7 (0.2%))

Final analyzed sample

(n=1166 (88.8%))

(men: n=473 (40.6%), women: n=693 (59.4%))

Excluded proxy interviews

(n=99 (7.1%))

# Table S1. Baseline characteristics by mobility mode among men and women aged 65+, Polish part of the COURAGE in Europe study (N = 1313), 2011. Unweighted data. Non-ambulatory group was not included in the main manuscript.

|  |  | **Men** | | | |  |  | **Women** | | | | | |
| --- | --- | --- | --- | --- | --- | --- | --- | --- | --- | --- | --- | --- | --- |
|  |  | Slow mobility users | | Fast mobility users  (n=83) | Non-ambulatory group  (n=37) | *p value^a^* | *p value^b^* | Slow mobility users | | Fast mobility users  (n=85) | Non-ambulatory group  (n=110) | *p value^a^* | *p value^b^* |
|  |  | Walkers  (n=292) | Cyclists  (n=98) |  |  |  |  | Walkers  (n=524) | Cyclists  (n=84) |  |  |  |  |
| **Mortality** [N(%)] | Alive | 125 (42.8) | 40 (40.8) | 42 (50.6) | 8 (21.6) | *0.030* | *0.362* | 271 (51.7) | 60 (71.4) | 51 (60.0) | 12 (10.9) | *<0.001* | *0.002* |
|  | Deceased | 167 (57.2) | 58 (59.2) | 41 (49.4) | 29 (78.4) |  |  | 253 (48.3) | 24 (28.6) | 34 (40.0) | 98 (89.1) |  |  |
| **Age** [Median (Q1;Q3) [Min-Max] | | 76 (70;81) [65-95] | 73.5 (69;79) [65-90] | 74 (68;79) [65-90] | 80 (74;84) [65-93] | *<0.001* | *0.024* | 75 (70;82)  [65-92] | 70 (67;73) [65-84] | 76 (70;81) [65-88] | 84 (80;87) [67-94] | *<0.001* | *<0.001* |
| **Married or cohabited** [N(%)] | | 194 (66.4) | 81 (82.7) | 61 (73.5) | 20 (54.1) | *0.003* | *0.008* | 163 (31.1) | 40 (47.6) | 34 (40.0) | 20 (18.2) | *<0.001* | *0.006* |
| **Level of education** [N(%)] | Priamary | 104 (35.6) | 45 (45.9) | 19 (22.9) | 18 (48.6) | *0.003* | *0.001* | 304 (58.0) | 43 (51.2) | 40 (47.1) | 86 (78.2) | *<0.001* | *0.120* |
|  | Vocational | 76 (26.0) | 27 (27.6) | 15 (18.1) | 9 (24.3) |  |  | 65 (12.4) | 15 (17.9) | 9 (10.6) | 5 (4.5) |  |  |
|  | High school | 65 (22.3) | 17 (17.3) | 27 (32.5) | 6 (16.2) |  |  | 99 (18.9) | 16 (19.0) | 27 (31.8) | 14 (12.7) |  |  |
|  | University | 47 (16.1) | 9 (9.2) | 22 (26.5) | 4 (10.8) |  |  | 56 (10.7) | 10 (11.9) | 9 (10.6) | 5 (4.5) |  |  |
| **Urban place of living** [N(%)] | | 180 (61.6) | 31 (31.6) | 46 (55.4) | 16 (43.2) | *<0.001* | *<0.001* | 310 (59.2) | 15 (17.9) | 60 (70.6) | 49 (44.5) | *<0.001* | *<0.001* |
| **Smoking** [N(%)] | Never smoked | 83 (28.4) | 33 (33.7) | 20 (24.1) | 16 (43.2) | *0.055* | *0.334* | 393 (75.0) | 71 (84.5) | 64 (75.3) | 99 (90) | *0.008* | *0.306* |
|  | Current smoker | 75 (25.7) | 22 (22.4) | 16 (19.3) | 2 (5.4) |  |  | 44 (8.4) | 6 (7.1) | 9 (10.6) | 1 (0.9) |  |  |
|  | Not current smoker | 134 (45.9) | 43 (43.9) | 47 (56.6) | 19 (51.4) |  |  | 87 (16.6) | 7 (8.3) | 12 (14.1) | 10 (9.1) |  |  |
| **Alcohol consumption** [N(%)] | Lifetime abstainers | 21 (7.2) | 8 (8.2) | 6 (7.2) | 6 (16.2) | *0.351* | *0.351* | 168 (32.1) | 30 (35.7) | 29 (34.1) | 45 (40.9) | *0.582* | *0.964* |
|  | Low risk drinkers | 244 (83.6) | 79 (80.6) | 72 (86.7) | 30 (81.1) |  |  | 348 (66.4) | 53 (63.1) | 55 (64.7) | 65 (59.1) |  |  |
|  | Infrequent high risk drinkers | 27 (9.2) | 11 (11.2) | 5 (6.0) | 1 (2.7) |  |  | 8 (1.5) | 1 (1.2) | 1 (1.2) | 0 (0.0) |  |  |
| **BMI** [Median (Q1;Q3)] | | 27.5 (24.4;30.1) | 27.3 (24.4;30.8) | 28.7 (26.3;31.1) | 25.7 (24.8;30.1) | *0.015* | *0.007* | 29.0 (25.5;32.4) | 30.1 (26.6;33.7) | 29.3 (26.0;33.1) | 29.1 (26.1;33.0) | *0.308* | *0.175* |
| **Physical activity** [N(%)] | High | 101 (34.7) | 50 (51.5) | 35 (42.2) | 2 (5.4) | *<0.001* | *0.008* | 198 (38.0) | 48 (57.1) | 42 (49.4) | 7 (6.4) | *<0.001* | *<0.001* |
|  | Moderate | 58 (19.9) | 23 (23.7) | 16 (19.3) | 1 (2.7) |  |  | 110 (21.1) | 22 (26.2) | 18 (21.2) | 12 (10.9) |  |  |
|  | Low | 132 (45.4) | 24 (24.7) | 32 (38.6) | 34 (91.9) |  |  | 213 (40.9) | 14 (16.7) | 25 (29.4) | 91 (82.7) |  |  |
| **Grip strength** [N(%)] | Normal | 163 (61.5) | 62 (68.9) | 58 (80.6) | 7 (36.8) | *<0.001* | *0.008* | 268 (60.4) | 58 (76.3) | 51 (66.2) | 19 (29.7) | *<0.001* | *0.024* |
| **Total number of diseases** [Median (Q1;Q3) [Mean (SD)]] | | 1 (0;2) | 1 (0;2) | 1 (0;2) | 2 (1;3) | *0.016* | *0.985* | 1 (1;2) | 1 (0;2) | 1 (1;2) | 2 (1;3) | *<0.001* | *0.047* |
| **Visual difficulty** [N(%)] | None | 168 (57.5) | 66 (67.3) | 62 (74.7) | 168 (57.5) | *0.006* | *0.022* | 292 (55.7) | 53 (63.1) | 52 (61.2) | 41 (37.3) | *<0.001* | *0.592* |
|  | Mild or moderate | 99 (33.9) | 23 (23.5) | 19 (22.9) | 99 (33.9) |  |  | 182 (34.7) | 26 (31.0) | 25 (29.4) | 42 (38.2) |  |  |
|  | Severe or extreme | 25 (8.6) | 9 (9.2) | 2 (2.4) | 25 (8.6) |  |  | 50 (9.5) | 5 (6.0) | 8 (9.4) | 27 (24.5) |  |  |
| **Hearing difficulty** [N(%)] | None | 160 (54.8) | 47 (48.0) | 49 (59.0) | 160 (54.8) | *0.528* | *0.522* | 327 (62.4) | 60 (71.4) | 50 (58.8) | 36 (32.7) | *<0.001* | *0.436* |
|  | Mild or moderate | 113 (38.7) | 41 (41.8) | 29 (34.9) | 113 (38.7) |  |  | 163 (31.1) | 21 (25.0) | 28 (32.9) | 54 (49.1) |  |  |
|  | Severe or extreme | 19 (6.5) | 10 (10.2) | 5 (6.0) | 19 (6.5) |  |  | 34 (6.5) | 3 (3.6) | 7 (8.2) | 20 (18.2) |  |  |
| **ADL scale** [Median (Q1;Q3) [Mean (SD)]] | | 0 (0;3) [2.2 (3.9)] | 0 (0;3) [2.1 (3.7)] | 0 (0;2) [1.6 (2.9)] | 9 (5;13) [8.7 (5)] | *<0.001* | *0.52* | 1 (0;6) [3.3 (4.7)] | 0 (0;2) [1.7 (2.9)] | 0 (0;3) [2.3 (3.6)] | 11 (6;14) [10.1 (4.7)] | *<0.001* | *<0.001* |
| **IADL scale** [Median (Q1;Q3) [Mean (SD)]] | | 0 (0;0) [0.7 (1.4)] | 0 (0;0) [0.4 (1)] | 0 (0;0) [0.3 (0.9)] | 4 (2;5) [3.3 (1.7)] | *<0.001* | *0.116* | 0 (0;1) [0.9 (1.5)] | 0 (0;0) [0.4 (0.9)] | 0 (0;0) [0.5 (1.1)] | 4 (2;5) [3.6 (1.7)] | *<0.001* | *0.002* |
| **Walking test** [Median (Q1;Q3)] | | 3.5 (2.9;5.0) | 3.6 (2.7;4.2) | 4.0 (2.9;5.4) | 2.8 (1.2;6.8) | *0.664* | *0.544* | 4.3 (3.0;5.9) | 4.0 (3.2;4.9) | 3.5 (3.0;5.0) | 5.3 (1.3;7.8) | *0.202* | *0.096* |
| **Perceived safety on the street** [N(%)] | None | 137 (46.9) | 55 (56.1) | 47 (56.6) | 137 (46.9) | *0.009* | *0.058* | 203 (38.7) | 44 (52.4) | 24 (28.2) | 34 (30.9) | *<0.001* | *0.003* |
|  | Mild or moderate | 101 (34.6) | 35 (35.7) | 28 (33.7) | 101 (34.6) |  |  | 156 (29.8) | 28 (33.3) | 29 (34.1) | 26 (23.6) |  |  |
|  | Severe or extreme | 54 (18.5) | 8 (8.2) | 8 (9.6) | 54 (18.5) |  |  | 165 (31.5) | 12 (14.3) | 32 (37.6) | 50 (45.5) |  |  |
| **Loneliness** [Median (Q1;Q3) [Mean (SD)]] | | 0.0 (0.0;33.3) [17.2 (24.3)] | 0.0 (0.0;16.7) [13.3 (21.3)] | 0.0 (0.0;16.7) [11.2 (18.4)] | 16.7 (0.0;50.0) [30.6 (35)] | *0.014* | *0.084* | 0.0 (0.0;33.3) [17.8 (24.1)] | 0.0 (0.0;16.7) [9.7 (16.8)] | 0.0 (0.0;33.3) [16.1 (22.5)] | 16.7 (0.0;50.0) [26.3 (27.8)] | *<0.001* | *0.022* |
| **Level of social networks** [Median (Q1;Q3)] | | 65.5 (56.1;72.7) | 67 (61.6;73.2) | 65.4 (55.1;73.8) | 61.1 (49.3;74.7) | *0.237* | *0.156* | 62.2 (54.7;70.0) | 66.4 (57.4;75.6) | 64.6 (53.7;71.7) | 59.8 (50.4;68.8) | *0.009* | *0.045* |
| **Social support** [Median (Q1;Q3)] | | 63.6 (45.5;72.7) | 72.7 (63.6;81.8) | 63.6 (54.6;72.7) | 59.1 (45.5;72.7) | *<0.001* | *<0.001* | 63.6 (54.6;72.7) | 68.2 (54.6;81.8) | 72.7 (54.6;81.8) | 63.6 (45.5;72.7) | *0.003* | *0.031* |
| **Trust in neighbors** [N(%)] | Great extent^1^ | 75 (25.7) | 33 (33.7) | 24 (28.9) | 14 (37.8) | *0.338* | *0.421* | 166 (31.7) | 27 (32.1) | 31 (36.5) | 32 (29.1) | *0.290* | *0.439* |
|  | Neither great nor small | 128 (43.8) | 39 (39.8) | 40 (48.2) | 11 (29.7) |  |  | 205 (39.1) | 31 (36.9) | 24 (28.2) | 35 (31.8) |  |  |
|  | Small extent^2^ | 89 (30.5) | 26 (26.5) | 19 (22.9) | 12 (32.4) |  |  | 153 (29.2) | 26 (31.0) | 30 (35.3) | 43 (39.1) |  |  |
| **Informal social participation** [Median (Q1;Q3)] | | 27.2 (17.8;39.3) | 26.6 (16.6;34.4) | 27.2 (23.9;43.8) | 11.0 (0.0;23.9) | *<0.001* | *0.080* | 27.2 (17.8;43.4) | 26.6 (20.4;43.8) | 27.0 (17.8;43.9) | 17.8 (0.0;24.7) | *<0.001* | *0.989* |
| **Formal social participation** [Median (Q1;Q3) [Mean (SD)]] | | 0.0 (0.0;26.4) [13.8 (17.9)] | 15.4 (0.0;28.8) [16.5 (17.7)] | 18.8 (0.0;34.0) [19.4 (18.8)] | 0.0 (0.0;0.0) [4.9 (14.1)] | *<0.001* | *0.020* | 0.0 (0.0;18.1) [10.3 (15.8)] | 0.0 (0.0;28.7) [13.8 (15.8)] | 13.7 (0.0;21.9) [14 (17.6)] | 0.0 (0.0;0.0) [0.8 (4.1)] | *<0.001* | *0.014* |
| **Religious Service Attendance** [N(%)] | Frequently | 106 (36.3) | 47 (48.0) | 32 (38.6) | 106 (36.3) | *<0.001* | *0.037* | 264 (50.4) | 51 (60.7) | 44 (51.8) | 7 (6.4) | *<0.001* | *0.019* |
|  | Ocasionally | 65 (22.3) | 26 (26.5) | 25 (30.1) | 65 (22.3) |  |  | 128 (24.4) | 26 (31.0) | 20 (23.5) | 15 (13.6) |  |  |
|  | Rarely | 121 (41.4) | 25 (25.5) | 26 (31.3) | 121 (41.4) |  |  | 132 (25.2) | 7 (8.3) | 21 (24.7) | 88 (80.0) |  |  |
| **COURAGE Built Environment Self-Reported Questionnaire (CBE-SR)** | | | | | | | | | | | | | |
| **Usability of the neighborhood environment** [Median (Q1;Q3)] | | 64.1 (48.2;89.7) | 52.9 (40.8;72.1) | 67.6 (52.3;84.9) | 53.8 (34.3;75.3) | *<0.001* | *0.001* | 61.4 (45.9;84.9) | 54.6 (39.6;80.6) | 72.2 (49.3;89.7) | 52.5 (34.3;72.2) | *<0.001* | *0.011* |
| **Hindrance of walkable environment** (*reversed*) [Median (Q1;Q3)] | | 61.8 (51.0;81.1) | 60.8 (47.2;81.1) | 68.9 (56.2;87.1) | 61.3 (55.5;76.9) | *0.305* | *0.172* | 64.2 (47.5;83.5) | 66.1 (50.6;82.3) | 65.1 (45.0;100.0) | 65.5 (50.7;87.1) | *0.557* | *0.533* |
| **Easiness of use of public buildings, places and facilities** [Median (Q1;Q3)] | | 57.6 (48.1;85.2) | 57.6 (48.1;86.9) | 66.3 (57.6;100.0) | 57.6 (48.2;75.9) | *0.196* | *0.119* | 57.6 (51.0;89.7) | 57.6 (48.1;85.5) | 57.6 (47.5;89.7) | 57.6 (30.2;77.5) | *0.098* | *0.604* |
| **Usability of the living place** [Median (Q1;Q3)] | | 63.0 (46.9;87.6) | 79.6 (52.4;93.1) | 76.3 (52.4;93.1) | 47.7 (44.6;81.1) | *<0.001* | *0.002* | 68.8 (48.8;93.1) | 75.1 (52.4;93.1) | 66.5 (47.3;93.1) | 61.0 (42.9;79.5) | *0.008* | *0.205* |
| **COURAGE Built Environment Outdoor Checklist (CBE-OUT)** | | | | | | | | | | | | | |
| **Streetscape** [Median (Q1;Q3)] | | 51.9 (45.7;56.8) | 50.6 (44.4;55.6) | 51.9 (46.9;56.8) | 50.6 (46.9;55.6) | *0.371* | *0.232* | 51.9 (46.9;58.0) | 50.6 (44.4;55.6) | 53.1 (46.9;59.3) | 51.9 (45.7;58.0) | *0.076* | *0.032* |
| **Walkways** [Median (Q1;Q3)] | | 62.2 (55.6;68.9) | 61.1 (55.6;66.7) | 62.2 (57.8;66.7) | 64.4 (56.1;71.1) | *0.255* | *0.219* | 62.2 (57.8;68.9) | 60.0 (53.3;66.7) | 62.2 (56.7;66.7) | 62.2 (56.7;66.7) | *0.229* | *0.118* |
| **Bikeways** [Median (Q1;Q3) [Mean (SD)]] | | 0.0 (0.0;0.0) [10.3 (25.5)] | 0.0 (0.0;0.0) [7.4 (22.6)] | 0.0 (0.0;0.0) [8.4 (24.9)] | 0.0 (0.0;0.0) [1.4 (8.3)] | *0.153* | *0.439* | 0.0 (0.0;0.0) [10.1 (25.6)] | 0.0 (0.0;0.0) [3.1 (14)] | 0.0 (0.0;0.0) [6.8 (19.6)] | 0.0 (0.0;0.0) [7.1 (21.1)] | *0.111* | *0.062* |
| **Street crossing/intersections** [Median (Q1;Q3)] | | 63.2 (0.0;67.8) | 0.0 (0.0;65.5) | 63.2 (0.0;67.8) | 60.9 (0;65.5) | *<0.001* | *<0.001* | 63.2 (0.0;67.8) | 56.3 (0.0;63.2) | 63.2 (0.0;67.8) | 60.9 (0.0;65.5) | *<0.001* | *0.001* |
| **Parking facilities** [Median (Q1;Q3)] | | 37.5 (25.0;37.5) | 37.5 (37.5;37.5) | 37.5 (37.5;37.5) | 37.5 (37.5;37.5) | *0.877* | *0.712* | 37.5 (25.0;37.5) | 37.5 (37.5;37.5) | 37.5 (25.0;37.5) | 37.5 (37.5;37.5) | *0.688* | *0.535* |
| **Public facilities and features of the street** [Median (Q1;Q3)] | | 40.0 (20.0;43.6) | 20.4 (20.0;40.0) | 33.4 (20.0;60.0) | 32.5 (20.0;40.0) | *0.115* | *0.056* | 40.0 (20.0;41.0) | 25.6 (20.0;40.0) | 40.0 (20.0;40.0) | 20.0 (20.0;40.0) | *<0.001* | *0.004* |
| **Land-use visible along the street/road** [Median (Q1;Q3)] | | 55.6 (44.4;72.2) | 50.0 (38.9;61.1) | 55.6 (44.4;66.7) | 44.4 (41.7;66.7) | *0.002* | *<0.001* | 55.6 (44.4;66.7) | 50.0 (38.9;61.1) | 50.0 (44.4;66.7) | 55.6 (44.4;66.7) | *0.002* | *<0.001* |
| **Site decay/urban blight** [Median (Q1;Q3)] | | 81.8 (77.4;90.9) | 81.8 (77.5;85.6) | 81.8 (78.7;85.5) | 83.0 (78.8;88.4) | *0.925* | *0.989* | 81.8 (75.2;86.0) | 81.8 (76.7;87.9) | 81.8 (75.6;87.7) | 81.8 (77.8;86.8) | *0.413* | *0.548* |

Note: ^a^ - p value for 4 groups, ^b^ - p value for 3 groups (without non-ambulatory), SD - Standard deviation; Q1 - first quartile; Q3 - third quartile; ^1^ - great = to a great or very great; ^2^ - small = to a small or very small; The mean and standard deviation were also provided where the differences in medians were barely noticeable

#

# Table S2. Baseline characteristics by mobility mode among men and women aged 65+, Polish part of the COURAGE in Europe study (N = 1313), 2011. Weighted data. Non-ambulatory group was not included in the main manuscript.

|  |  | | **Men** | | | |  |  | **Women** | | | | | |
| --- | --- | --- | --- | --- | --- | --- | --- | --- | --- | --- | --- | --- | --- | --- |
|  |  | | Slow mobility users | | Fast mobility users | Non-ambulatory group | *p value^a^* | *p value^b^* | Slow mobility users | | Fast mobility users | Non-ambulatory group | *p value^a^* | *p value^b^* |
|  |  |  | Walkers | Cyclists |  |  |  |  | Walkers | Cyclists |  |  |  |  |
| **Mortality** (%) | Alive | | 47.6 | 51.1 | 48.3 | 19.4 | *0.032* | *0.851* | 60.5 | 75.3 | 69.2 | 9.4 | *<0.001* | *0.032* |
|  | Deceased | | 52.4 | 48.9 | 51.7 | 80.6 |  |  | 39.5 | 24.7 | 30.8 | 90.6 |  |  |
| **Age** [Median (Q1;Q3) | | | 72 (68;78) | 73 (69;76) | 73 (69;78) | 76 (71;84) | *0.049* | *0.731* | 74 (69;80) | 69 (66;72) | 74 (69;81) | 84 (78;87) | *<0.001* | *<0.001* |
| **Married or cohabited** (%) | | | 79.4 | 89.9 | 85.3 | 65.6% | *0.017* | *0.061* | 42.9 | 60.8 | 48.3 | 22.4 | *<0.001* | *0.027* |
| **Level of education** (%) | Priamary | | 31.8 | 43.9 | 14.1 | 54.9 | *<0.001* | *0.001* | 51.4 | 48.4 | 38.0 | 70.6 | *0.014* | *0.421* |
|  | Vocational | | 28.0 | 28.5 | 20.7 | 20.6 |  |  | 14.1 | 14.8 | 17.8 | 3.4 |  |  |
|  | High school | | 23.5 | 13.7 | 37.3 | 10.8 |  |  | 23.6 | 22.1 | 31.6 | 18.6 |  |  |
|  | University | | 16.7 | 13.9 | 28.0 | 13.8 |  |  | 11.0 | 14.7 | 12.6 | 7.4 |  |  |
| **Urban place of living** (%) | | | 69.9 | 42.6 | 76.7 | 58.0% | *<0.001* | *<0.001* | 70.8 | 33.8 | 77.7 | 56.8 | *<0.001* | *<0.001* |
| **Smoking** (%) | Never smoked | | 28.1 | 29.1 | 25.1 | 35.0 | *0.066* | *0.222* | 74.6 | 82.0 | 71.6 | 86.2 | *0.136* | *0.507* |
|  | Current smoker | | 28.3 | 17.8 | 21.2 | 4.8 |  |  | 6.9 | 5.2 | 10.3 | 1.1 |  |  |
|  | Not current smoker | | 43.6 | 53.1 | 53.7 | 60.2 |  |  | 18.6 | 12.8 | 18.1 | 12.6 |  |  |
| **Alcohol consumption** (%) | Lifetime abstainers | | 5.0 | 5.2 | 7.4 | 12.3 | *0.158* | *0.189* | 27.8 | 26.8 | 25.9 | 38.9 | *0.470* | *0.981* |
|  | Low risk drinkers | | 80.8 | 83.7 | 88.5 | 85.0 |  |  | 71.1 | 72.0 | 73.2 | 61.1 |  |  |
|  | Infrequent high risk drinkers | | 14.2 | 11.1 | 4.1 | 2.8 |  |  | 1.1 | 1.3 | 0.9 | 0.0 |  |  |
| **BMI** [Median (Q1;Q3)] | | | 27.9 (24.7;30.4) | 28.7 (24.9;31.9) | 30.1 (27.7;31.2) | 25.7 (24.9;30.1) | *<0.001* | *<0.001* | 28.8 (25.7;32.4) | 29.8 (25.5;33.5) | 28.6 (26.0;32.9) | 29.3 (25.4;33.2) | *0.984* | *0.932* |
| **Physical activity** (%) | High | | 42.4 | 54.9 | 42.5 | 5.4 | *<0.001* | *0.073* | 40.7 | 64.3 | 51.2 | 6.7 | *<0.001* | *<0.001* |
|  | Moderate | | 17.0 | 21.0 | 21.9 | 6.7 |  |  | 22.8 | 23.5 | 21.6 | 8.7 |  |  |
|  | Low | | 40.6 | 24.1 | 35.6 | 87.9 |  |  | 36.6 | 12.2 | 27.2 | 84.6 |  |  |
| **Grip strength** (%) | Normal | | 71.8 | 77.3 | 76.5 | 52.4 | *0.292* | *0.548* | 59.9 | 84.9 | 66.4 | 33.8 | *<0.001* | *<0.001* |
| **Total number of diseases** [Median (Q1;Q3) [Mean (SD)]] | | | 1 (0;2)  [1.2 (1.1)] | 1 (0;2)  [1.2 (1.2)] | 1 (0;2)  [1.3 (1.4)] | 2 (1;3) [1.9 (1.4)] | *0.074* | *0.958* | 1 (1;2)  [1.6 (1.3)] | 1 (0;2)  [1.2 (1.2)] | 1 (1;2)  [1.5 (1.3)] | 2 (1;3) | *<0.001* | *0.020* |
| **Visual difficulty** (%) | None | | 64.8 | 76.1 | 73.7 | 46.6 | *0.002* | *0.006* | 58.2 | 65.1 | 68.7 | 43.2 | *<0.001* | *0.100* |
|  | Mild or moderate | | 29.3 | 13.2 | 24.5 | 39.9 |  |  | 33.7 | 32.5 | 22.6 | 38.0 |  |  |
|  | Severe or extreme | | 5.9 | 10.7 | 1.8 | 13.4 |  |  | 8.1 | 2.4 | 8.7 | 18.8 |  |  |
| **Hearing difficulty** (%) | None | | 63.0 | 51.4 | 55.1 | 51.9 | *0.435* | *0.289* | 66.4 | 74.3 | 60.2 | 35.6 | *<0.001* | *0.443* |
|  | Mild or moderate | | 30.6 | 41.3 | 37.1 | 38.0 |  |  | 28.2 | 24.0 | 33.5 | 49.7 |  |  |
|  | Severe or extreme | | 6.4 | 7.3 | 7.7 | 10.1 |  |  | 5.4 | 1.6 | 6.3 | 14.8 |  |  |
| **ADL scale** [Median (Q1;Q3) [Mean (SD)]] | | | 0 (0;2)  [1.8 (3.2)] | 0 (0;3)  [1.8 (2.9)] | 0 (0;2)  [1.6 (2.7)] | 10 (7;14) [9.8 (4.7)] | *<0.001* | *0.906* | 1 (0;6)  [3.3 (4.0)] | 0 (0;2)  [1.7 (2.9)] | 0 (0;4)  [2.5 (3.5)] | 11 (7;14) [10.2 (4.7)] | *<0.001* | *<0.001* |
| **IADL scale** [Median (Q1;Q3) [Mean (SD)]] | | | 0 (0;0)  [0.6 (1.4)] | 0 (0;0)  [0.4 (0.8)] | 0 (0;0)  [0.3 (0.8)] | 4 (2;5) [3.5 (1.4)] | *<0.001* | *0.274* | 0 (0;1)  [0.8 (1.4)] | 0 (0;0)  [0.4 (1.1)] | 0 (0;1)  [0.6 (1.2)] | 4 (2;5)  [3.6 (1.6)] | *<0.001* | *0.066* |
| **Walking test** [Median (Q1;Q3)] | | | 3.6 (3.0;4.0) | 3.5 (2.7;4.0) | 4.0 (2.6;5.0) | 1.2 (1.0;6.8) | *0.257* | *0.672* | 4.4 (3.1;6.0) | 3.7 (3.2;4.6) | 3.5 (3.1;5.0) | 5.5 (1.4;6.0) | *0.020* | *0.086* |
| **Perceived safety on the street** (%) | None | | 51.0 | 48.2 | 54.3 | 43.5 | *0.254* | *0.351* | 38.4 | 51.8 | 25.0 | 30.7 | *<0.001* | *<0.001* |
|  | Mild or moderate | | 30.9 | 40.0 | 31.6 | 25.7 |  |  | 28.8 | 39.4 | 38.4 | 23.0 |  |  |
|  | Severe or extreme | | 18.1 | 11.7 | 14.1 | 30.8 |  |  | 32.8 | 8.8 | 36.6 | 46.4 |  |  |
| **Loneliness** [Median (Q1;Q3) [Mean (SD)]] | | | 0.0 (0.0;16.7)  [14.4 (20.8)] | 0.0 (0.0;16.7)  [12.6 (20.3)] | 0.0 (0.0;0.0)  [6.2 (14.2)] | 17.0 (0.0;50.0) [26.5 (32.8)] | *<0.001* | *<0.001* | 0.0 (0.0;33.3) [16.7 (22.9)] | 0.0 (0.0;16.7) [9.9 (18.6)] | 0.0 (0.0;16.7) [11.3 (19.7)] | 17.0 (0.0;50.0) [29.1 (31.8)] | *<0.001* | *0.007* |
| **Level of social networks** [Median (Q1;Q3)] | | | 68.9 (58.5;74.7) | 65.7 (56.1;71.6) | 61.8 (55.1;73.5) | 60.6 (49.8;72.6) | *0.016* | *0.037* | 62.1 (54.8;70.2) | 68.5 (57.9;77.6) | 64.9 (55.5;70.4) | 59.2 (48.9;68.9) | *0.008* | *0.017* |
| **Social support** [Median (Q1;Q3)] | | | 63.6 (54.6;72.7) | 72.7 (63.6;81.8) | 63.6 (45.5;72.7) | 54.6 (45.5;72.7) | *<0.001* | *<0.001* | 63.6 (54.5;72.7) | 72.7 (54.5;81.8) | 72.7 (54.5;81.8) | 63.6 (45.5;72.7) | *0.003* | *0.009* |
| **Trust in neighbors** (%) | Great extent^1^ | | 29.0 | 30.6 | 29.0 | 34.0 | *0.741* | *0.511* | 30.8 | 27.1 | 33.7 | 29.8 | *0.032* | *0.239* |
|  | Neither great nor small | | 37.8 | 44.3 | 45.1 | 37.7 |  |  | 41.3 | 43.2 | 29.1 | 26.7 |  |  |
|  | Small extent^2^ | | 33.2 | 25.2 | 26.0 | 28.3 |  |  | 27.9 | 29.8 | 37.2 | 43.5 |  |  |
| **Informal social participation** [Median (Q1;Q3)] | | | 27.2 (19.0;41.1) | 26.6 (14.3;37.0) | 27.2 (21.2;43.9) | 14.8 (0.0;29.2) | *<0.001* | *0.096* | 27.2 (21.2;43.2) | 26.6 (21.2;43.2) | 28.8 (17.8;46.5) | 14.3 (0.0;24.9) | *<0.001* | *0.360* |
| **Formal social participation** [Median (Q1;Q3) [Mean (SD)]] | | | 0.0  (0.0;26.4) [14.0 (17.3)] | 0.0 (0.0;28.8) [14.5 (17.7)] | 15.4 (0.0;28.8) [18.2 (19.7)] | 0.0 (0.0;0.0) [3.52 (12.4)] | *<0.001* | *0.225* | 0.0 (0.0;17.0) [9.8 (14.6)] | 0.0 (0.0;28.8) [12.9 (16.4)] | 0.0 (0.0;24.5) [15.5 (20.1)] | 0.0 (0.0;0.0) [0.8 (4.3)] | *<0.001* | *0.005* |
| **Religious Service Attendance (%)** | | Frequently | 38.6 | 45.2 | 34.4 | 3.7 | *<0.001* | *0.257* | 48.4 | 61.1 | 54.4 | 3.5 | *<0.001* | *0.005* |
|  |  | Ocasionally | 22.6 | 26.7 | 28.3 | 16.7 |  |  | 23.8 | 32.8 | 26.1 | 14.2 |  |  |
|  |  | Rarely | 38.8 | 28.1 | 37.3 | 79.6 |  |  | 27.8 | 6.1 | 19.5 | 82.3 |  |  |
| **COURAGE Built Environment Self-Reported Questionnaire (CBE-SR)** | | | | | | | | | | | | | | |
| **Usability of the neighborhood environment** [Median (Q1;Q3)] | | | 70.4 (55.1;89.7) | 56.2 (47.5;83.9) | 72.1 (59.3;100.0) | 62.7 (49.6;89.7) | *<0.001* | *<0.001* | 68.2 (53.8;89.7) | 59.3 (48.9;84.9) | 78.8 (56.2;100.0) | 56.2 (40.3;75.3) | *0.004* | *0.199* |
| **Hindrance of walkable environment** (*reversed*) [Median (Q1;Q3)] | | | 62.2 (46.8;77.2) | 54.2 (45.0;76.9) | 64.1 (54.4;100) | 60.6 (40.0;71.5) | *0.111* | *0.056* | 63.3 (44.8;82.2) | 66.0 (50.2;83.5) | 66.0 (51.2;100.0) | 70.2 (53.1;87.1) | *0.040* | *0.029* |
| **Easiness of use of public buildings, places and facilities** [Median (Q1;Q3)] | | | 62.4 (57.6;85.5) | 57.6 (52.2;85.5) | 71.3 (57.6;100) | 61.2 (57.6;85.5) | *0.005* | *0.002* | 57.6 (55.8;100.0) | 57.6 (48.1;100.0) | 61.2 (48.1;100.0) | 57.6 (36.1;96.6) | *0.786* | *0.851* |
| **Usability of the living place** [Median (Q1;Q3)] | | | 67.7 (47.7;88.1) | 79.6 (52.1;93.1) | 73.7 (52.4;85.4) | 46.9 (27.4;88.1) | *0.038* | *0.068* | 70.7 (48.8;93.1) | 76.2 (52.1;93.1) | 70.7 (48.9;95.0) | 52.4 (43.5;79.5) | *0.036* | *0.574* |
| **COURAGE Built Environment Outdoor Checklist (CBE-OUT)** | | | | | | | | | | | | | | |
| **Streetscape** [Median (Q1;Q3)] | | | 51.9 (45.7;58.0) | 51.9 (44.4;56.8) | 53.1 (48.1;60.5) | 50.6 (46.9;55.6) | *0.041* | *0.017* | 53.1 (46.9;59.3) | 50.6 (45.7;58.0) | 54.3 (49.4;61.7) | 53.1 (46.9;59.3) | *0.027* | *0.011* |
| **Walkways** [Median (Q1;Q3)] | | | 60.0 (53.3;68.9) | 62.2 (57.8;66.7) | 66.7 (60;68.9) | 64.4 (57.8;71.1) | *0.006* | *0.009* | 64.4 (57.8;68.9) | 60.0 (57.8;68.9) | 62.2 (56.7;68.9) | 62.2 (57.8;66.7) | *0.791* | *0.807* |
| **Bikeways** [Median (Q1;Q3) [Mean (SD)]] | | | 0.0 (0.0;0.0) [9.7 (24.1)] | 0.0 (0.0;0.0) [12.8 (29.3)] | 0.0 (0.0;0.0) [9.0 (24.5)] | 0.0 (0.0;0.0) [3.5 (12.9)] | *0.571* | *0.715* | 0.0 (0.0;0.0) [9.1 (24.5)] | 0.0 (0.0;0.0) [5.6 (19.8)] | 0.0 (0.0;0.0) [8.1 (22.4)] | 0.0 (0.0;0.0) [8.3 (21.4)] | *0.595* | *0.418* |
| **Street crossing/intersections** [Median (Q1;Q3)] | | | 63.2 (54.0;67.8) | 56.3 (0.0;67.8) | 65.5 (54.0;70.1) | 63.2 (0.0;65.5) | *0.003* | *<0.001* | 63.2 (49.4;67.8) | 58.6 (0.0;65.5) | 65.5 (0.0;72.4) | 63.2 (0.0;65.5) | *<0.001* | *0.004* |
| **Parking facilities** [Median (Q1;Q3)] | | | 37.5 (25.0;37.5) | 37.5 (37.5;37.5) | 37.5 (37.5;37.5) | 37.5 (37.5;37.5) | *0.352* | *0.202* | 37.5 (25.0;37.5) | 37.5 (37.5;37.5) | 37.5 (37.5;50.0) | 37.5 (37.5;37.5) | *0.302* | *0.177* |
| **Public facilities and features of the street** [Median (Q1;Q3)] | | | 40.0 (20.0;50.0) | 31.9 (20.0;40.0) | 40.0 (20.0;60.0) | 40.0 (20.0;50.0) | *0.057* | *0.031* | 40.0 (20.0;50.0) | 40.0 (20.0;40.0) | 40.0 (40.0;60.0) | 20.0 (20.0;50.0) | *0.001* | *0.024* |
| **Land-use visible along the street/road** [Median (Q1;Q3)] | | | 61.1 (50.0;72.2) | 50.0 (38.9;61.1) | 61.1 (55.6;72.2) | 61.1 (44.4;72.2) | *0.001* | *<0.001* | 61.1 (50.0;72.2) | 55.6 (44.4;66.7) | 55.6 (50.0;66.7) | 55.6 (44.4;72.2) | *0.009* | *0.007* |
| **Site decay/urban blight** [Median (Q1;Q3)] | | | 81.8 (74.4;87.0) | 81.8 (74.8;85.7) | 80.6 (75.4;85.1) | 83.0 (78.8;87.7) | *0.675* | *0.752* | 81.8 (74.5;86.3) | 81.6 (72.7;86.6) | 81.5 (74.5;84.9) | 81.8 (77.1;90.9) | *0.512* | *0.674* |

Note: ^a^ - p value for 4 groups, ^b^ - p value for 3 groups (without non-ambulatory), SD - Standard deviation; Q1 - first quartile; Q3 - third quartile; ^1^ - great = to a great or very great; ^2^ - small = to a small or very small; The mean and standard deviation were also provided where the differences in medians were barely noticeable

# Figure S3. Assumed causal model. Diagram of the assumed associations between built environment on survival time through the potential social participation mediators.

Formal/informal social participation

Religious services attendance

Built environment

Survival time

#

# Table S3. Results of mediation analysis: The effect of built environment on survival time through formal social participation (N = 1166). Weighted data.

|  | Effect Type | **MEN** | | | **WOMEN** | | |
| --- | --- | --- | --- | --- | --- | --- | --- |
|  |  | **Slow mobility users** | | **Fast mobility users**  Estimate [Q1;Q3] | **Slow mobility users** | | **Fast mobility users**  Estimate [Q1;Q3] |
|  |  | Walkers  Estimate [Q1;Q3] | Cyclists  Estimate [Q1;Q3] |  | Walkers  Estimate [Q1;Q3] | Cyclists  Estimate [Q1;Q3] |  |
| **COURAGE Built Environment Self-Reported Questionnaire (CBE-SR)** | | | | | | | |
| Hindrance of walkable environment  (*reversed*) | ACME | 1.00 [0.99;1.02] | 1.00 [0.95;1.04] | 1.08 [0.98;1.28] | 0.97 [0.92;1.00] | 1.00 [0.89;1.14] | 0.97 [0.85;1.04] |
|  | ADE | 1.05 [0.93;1.11] | 0.98 [0.53;1.17] | 0.83 [0.44;1.08] | 0.98 [0.76;1.14] | 1.05 [0.22;1.57] | 1.21 [0.85;1.51] |
|  | TE | 1.05 [0.94;1.11] | 0.98 [0.54;1.17] | 0.90 [0.49;1.11] | 0.96 [0.74;1.12] | 1.05 [0.23;1.57] | 1.18 [0.79;1.42] |
| **COURAGE Built Environment Outdoor Checklist (CBE-OUT)** | | | | | | | |
| Streetscape | ACME | 1.01 [0.98;1.03] | 0.93 [0.65;1.03] | 0.78 [0.26;1.03] | 0.99 [0.92;1.03] | 0.96 [0.69;1.19] | 1.61 [0.71;48.43] |
|  | ADE | 1.10** [1.06;1.15] | 0.97 [0.26;1.35] | 0.88 [0.04;1.56] | 1.19 [0.92;1.29] | 0.65 [0.01;1.8] | 0.01 [0.00;1.27] |
|  | TE | 1.11** [1.06;1.14] | 0.90 [0.21;1.23] | 0.69 [0.02;1.19] | 1.17 [0.91;1.26] | 0.63 [0.01;1.61] | 0.02 [0.00;1.36] |
| Walkways | ACME | 1.00 [0.99;1.01] | 1.01 [0.98;1.05] | 74.02 [0.00;>1000] | 0.89 [0.65;1.02] | 2.24 [0;>1000] | 0.99 [0.78;1.23] |
|  | ADE | 1.09*** [1.06;1.13] | 1.07 [0.99;1.16] | 0.00*** [0.00;0.01] | 0.63 [0.08;1.20] | 0.00 [0.00;1.41] | 0.97 [0.21;1.43] |
|  | TE | 1.09*** [1.06;1.12] | 1.08 [1.01;1.16] | 0.00*** [0.00;0.02] | 0.56 [0.06;1.15] | 0.00 [0.00;1.42] | 0.96 [0.18;1.35] |
| Bikeways | ACME | 1.01 [1.00;1.02] | 1.01 [0.98;1.05] | 1.00 [0.94;1.06] | 1.01 [0.98;1.05] | 0.00 [0.00;>1000] | 1.40 [0.94;2.85] |
|  | ADE | 1.04 [0.92;1.16] | 1.30 [0.97;1.74] | 0.95 [0.73;1.19] | 1.12 [0.94;1.32] | >1000 [0.00;>1000] | 0.50 [0.12;1.20] |
|  | TE | 1.05 [0.93;1.17] | 1.31 [0.98;1.76] | 0.95 [0.72;1.20] | 1.13 [0.95;1.35] | >1000 [0.00;>1000] | 0.70 [0.22;1.40] |
| Street crossing/intersections | ACME | 1.00 [0.99;1.01] | 1.00 [0.97;1.02] | 0.98 [0.9;1.010] | 0.99 [0.96;1.02] | 0.98 [0.66;1.36] | 1.03 [0.96;1.18] |
|  | ADE | 1.08* [1.02;1.13] | 1.21** [1.07;1.38] | 1.03 [0.79;1.17] | 1.03 [0.89;1.14] | 0.67 [0.07;1.61] | 1.10 [0.70;1.40] |
|  | TE | 1.08* [1.01;1.12] | 1.21** [1.07;1.37] | 1.01 [0.77;1.14] | 1.02 [0.88;1.13] | 0.66 [0.06;1.51] | 1.13 [0.72;1.46] |
| Public facilities and features of the street | ACME | 1.01 [0.99;1.02] | 0.94 [0.81;1.01] | 0.86 [0.49;1.24] | 1.06** [1.02;1.13] | 0.98 [0.71;1.25] | 1.05 [0.90;1.32] |
|  | ADE | 1.11* [1.00;1.19] | 1.20 [0.86;1.49] | 0.21** [0.01;0.86] | 1.12 [0.91;1.26] | 1.02 [0.08;2.25] | 1.24 [0.75;1.57] |
|  | TE | 1.12* [1.01;1.19] | 1.14 [0.78;1.35] | 0.18*** [0.01;0.81] | 1.20 [0.99;1.33] | 1.00 [0.06;2.18] | 1.30 [0.85;1.60] |
| Note: ACME – average casual mediation effect, ADE – average direct effect, TE - Total effect; * - p value <0.05; **- p value <0.01; *** - p value < 0.001; Q1 - first quartile; Q3 - third quartile. Direct, indirect and total effects are exponentiated.  The models with significate ACME were further adjusted for age (and then additionally for marital status, level of education, place of living, BMI, physical activity, grip strength, total number of diseases, visual difficulties, ADL, IADL, perceived safety on the street; then additionally adjusted for loneliness, social support, social network) and the findings were not statistically significant. | | | | | | | |

#

# Table S4. Results of mediation analysis: The effect of built environment on survival time through informal social participation (N = 1166). Weighted data.

|  | Effect Type | **MEN** | | | **WOMEN** | | |
| --- | --- | --- | --- | --- | --- | --- | --- |
|  |  | **Slow mobility users** | | **Fast mobility users**  Estimate [Q1;Q3] | **Slow mobility users** | | **Fast mobility users**  Estimate [Q1;Q3] |
|  |  | Walkers  Estimate [Q1;Q3] | Cyclists  Estimate [Q1;Q3] |  | Walkers  Estimate [Q1;Q3] | Cyclists  Estimate [Q1;Q3] |  |
| **COURAGE Built Environment Self-Reported Questionnaire (CBE-SR)** | | | | | | | |
| Hindrance of walkable environment  (*reversed*) | ACME | 1.00 [0.99;1.02] | 0.98 [0.88;1.04] | 1.12* [1.00;1.45] | 0.97 [0.92;1.01] | 0.97 [0.75;1.08] | 1.00 [0.93;1.07] |
|  | ADE | 1.05 [0.95;1.11] | 1.00 [0.62;1.20] | 0.75 [0.33;1.05] | 0.97 [0.74;1.13] | 1.11 [0.31;1.71] | 1.18 [0.79;1.40] |
|  | TE | 1.06 [0.96;1.11] | 0.98 [0.60;1.19] | 0.84 [0.41;1.10] | 0.94 [0.70;1.11] | 1.07 [0.29;1.60] | 1.18 [0.79;1.39] |
| **COURAGE Built Environment Outdoor Checklist (CBE-OUT)** | | | | | | | |
| Streetscape | ACME | 1.00 [0.99;1.01] | 1.05 [0.86;1.38] | 1.03 [0.81;1.33] | 0.99 [0.91;1.04] | 1.01 [0.79;1.29] | 0.89 [0.18;2.58] |
|  | ADE | 1.11** [1.06;1.15] | 0.85 [0.18;1.21] | 0.77 [0.05;1.20] | 1.20 [1.00;1.32] | 0.88 [0.09;1.67] | 0.05 [0.00;1.36] |
|  | TE | 1.11** [1.07;1.14] | 0.89 [0.20;1.20] | 0.80 [0.06;1.18] | 1.19 [0.98;1.27] | 0.89 [0.07;1.62] | 0.04 [0.00;1.33] |
| Walkways | ACME | 1.00 [0.99;1.01] | 1.00 [0.98;1.03] | 902.37 [0;>1000] | 0.92 [0.65;1.09] | 28.37 [0.00;>1000] | 0.99 [0.73;1.18] |
|  | ADE | 1.09** [1.05;1.13] | 1.07* [1.01;1.16] | 0.00*** [0.00;0.01] | 0.61 [0.09;1.19] | 0.00 [0.00;1.41] | 0.92 [0.12;1.39] |
|  | TE | 1.09** [1.05;1.12] | 1.07* [1.02;1.15] | 0.00*** [0.00;0.03] | 0.56 [0.06;1.16] | 0.00 [0.00;1.40] | 0.91 [0.16;1.30] |
| Bikeways | ACME | 1.00 [0.99;1.01] | 1.00 [0.95;1.06] | 0.95 [0.85;1.01] | 0.94** [0.88;0.98] | >1000 [0.00;>1000] | 1.01 [0.80;1.24] |
|  | ADE | 1.05 [0.94;1.18] | 1.30 [0.98;1.79] | 0.98 [0.75;1.25] | 1.18 [0.97;1.41] | >1000 [0.00;>1000] | 0.78 [0.35;1.38] |
|  | TE | 1.05 [0.94;1.18] | 1.30 [0.98;1.77] | 0.93 [0.70;1.19] | 1.12 [0.92;1.33] | >1000 [0.00;>1000] | 0.79 [0.36;1.36] |
| Street crossing/intersections | ACME | 1.00 [0.99;1.01] | 1.00 [0.96;1.03] | 1.03 [1.00;1.11] | 0.97 [0.93;1.01] | 1.01 [0.85;1.22] | 0.99 [0.93;1.04] |
|  | ADE | 1.07* [1.00;1.12] | 1.21** [1.06;1.36] | 0.98 [0.76;1.12] | 1.05 [0.90;1.17] | 0.59 [0.05;1.44] | 1.13 [0.76;1.41] |
|  | TE | 1.07* [1.00;1.12] | 1.21** [1.07;1.34] | 1.01 [0.79;1.14] | 1.02 [0.86;1.15] | 0.60 [0.05;1.45] | 1.12 [0.74;1.40] |
| Public facilities and features of the street | ACME | 1.00 [0.99;1.01] | 1.01 [0.89;1.17] | 0.91 [0.62;1.11] | 1.05* [1.01;1.12] | 1.08 [0.78;2.00] | 0.97 [0.79;1.08] |
|  | ADE | 1.12* [1.01;1.19] | 1.12 [0.79;1.36] | 0.19** [0.01;0.80] | 1.15 [0.94;1.29] | 0.92 [0.03;2.05] | 1.32 [0.88;1.67] |
|  | TE | 1.12* [1.01;1.19] | 1.13 [0.84;1.32] | 0.17** [0.01;0.80] | 1.21* [1.02;1.34] | 0.99 [0.04;2.21] | 1.29 [0.85;1.55] |
| Note: ACME – average casual mediation effect, ADE – average direct effect, TE - Total effect; * - p value <0.05; **- p value <0.01; *** - p value < 0.001; Q1 - first quartile; Q3 - third quartile. Direct, indirect and total effects are exponentiated. | | | | | | | |

#

# Table S5. Results of mediation analysis: The effect of built environment on survival time through religious services attendance (N = 1166). Weighted data.

|  | Effect Type | **MEN** | | | **WOMEN** | | |
| --- | --- | --- | --- | --- | --- | --- | --- |
|  |  | **Slow mobility users** | | **Fast mobility users**  Estimate [Q1;Q3] | **Slow mobility users** | | **Fast mobility users**  Estimate [Q1;Q3] |
|  |  | Walkers  Estimate [Q1;Q3] | Cyclists  Estimate [Q1;Q3] |  | Walkers  Estimate [Q1;Q3] | Cyclists  Estimate [Q1;Q3] |  |
| **COURAGE Built Environment Self-Reported Questionnaire (CBE-SR)** | | | | | | | |
| Hindrance of walkable environment  (*reversed*) | ACME | 1.01 [1.00;1.04] | 1.02 [0.94;1.16] | 1.02 [0.95;1.11] | 0.99 [0.97;1.02] | 0.95 [0.67;1.07] | 1.03 [0.97;1.16] |
|  | ADE | 1.04 [0.93;1.10] | 0.96 [0.51;1.16] | 0.88 [0.48;1.10] | 0.95 [0.74;1.10] | 1.13 [0.31;1.78] | 1.17 [0.86;1.38] |
|  | TE | 1.05 [0.95;1.11] | 0.98 [0.58;1.17] | 0.89 [0.50;1.10] | 0.95 [0.74;1.09] | 1.08 [0.26;1.59] | 1.20 [0.90;1.43] |
| **COURAGE Built Environment Outdoor Checklist (CBE-OUT)** | | | | | | | |
| Streetscape | ACME | 1.01 [1.00;1.03] | 0.93 [0.61;1.11] | 0.88 [0.40;1.25] | 1.00 [0.96;1.04] | 1.04 [0.49;2.20] | 1.21 [0.23;8.88] |
|  | ADE | 1.10** [1.06;1.13] | 0.97 [0.32;1.34] | 0.78 [0.05;1.36] | 1.16 [0.96;1.25] | 0.34 [0.00;1.86] | 0.01 [0.00;1.37] |
|  | TE | 1.11*** [1.06;1.14] | 0.91 [0.27;1.21] | 0.68 [0.02;1.16] | 1.17 [0.96;1.25] | 0.36 [0.00;1.83] | 0.01 [0.00;1.39] |
| Walkways | ACME | 1.01 [1.00;1.02] | 1.00 [0.98;1.05] | 0.10 [0.00;>1000] | 0.99 [0.87;1.11] | 0.00 [0.00;>1000] | 1.06 [0.91;1.63] |
|  | ADE | 1.09*** [1.05;1.12] | 1.07* [1.01;1.16] | 0.00*** [0.00;0.01] | 0.60 [0.09;1.15] | 0.00 [0.00;1.75] | 0.92 [0.14;1.36] |
|  | TE | 1.09*** [1.06;1.12] | 1.07 [1.01;1.16] | 0.00*** [0.00;0.01] | 0.59 [0.08;1.15] | 0.00 [0.00;1.53] | 0.98 [0.28;1.40] |
| Bikeways | ACME | 0.99 [0.96;1.00] | 0.99 [0.94;1.02] | 0.99 [0.94;1.03] | 0.96** [0.92;0.99] | 0.00 [0.00;>1000] | 0.88 [0.62;1.03] |
|  | ADE | 1.07 [0.94;1.19] | 1.32 [1.00;1.79] | 0.95 [0.73;1.18] | 1.17 [0.98;1.38] | >1000 [0.00;>1000] | 0.89 [0.40;1.76] |
|  | TE | 1.05 [0.93;1.18] | 1.31 [0.99;1.76] | 0.94 [0.73;1.18] | 1.13 [0.95;1.33] | >1000 [0.00;>1000] | 0.79 [0.34;1.51] |
| Street crossing/intersections | ACME | 1.00 [0.99;1.01] | 0.99 [0.95;1.01] | 1.00 [0.97;1.03] | 0.98 [0.96;1.00] | 0.92 [0.51;1.25] | 1.00 [0.88;1.12] |
|  | ADE | 1.08* [1.02;1.13] | 1.22*** [1.09;1.37] | 1.00 [0.75;1.14] | 1.05 [0.92;1.15] | 0.53 [0.02;1.63] | 1.13 [0.74;1.48] |
|  | TE | 1.08* [1.02;1.13] | 1.21*** [1.08;1.35] | 1.00 [0.74;1.14] | 1.03 [0.9;1.14] | 0.49 [0.02;1.60] | 1.14 [0.73;1.49] |
| Public facilities and features of the street | ACME | 1.02 [1.00;1.04] | 0.96 [0.84;1.04] | 1.00 [0.84;1.2] | 0.98 [0.94;1.00] | 1.00 [0.41;1.92] | 0.90 [0.63;1.02] |
|  | ADE | 1.10 [0.98;1.18] | 1.17 [0.84;1.42] | 0.21** [0.01;0.83] | 1.22* [1.02;1.34] | 0.85 [0.01;2.62] | 1.47* [1.02;2.18] |
|  | TE | 1.12 [1.00;1.20] | 1.13 [0.77;1.33] | 0.21** [0.01;0.83] | 1.19 [0.99;1.32] | 0.85 [0.01;2.61] | 1.32 [0.85;1.73] |
| Note: ACME – average casual mediation effect, ADE – average direct effect, TE - Total effect; * - p value <0.05; **- p value <0.01; *** - p value < 0.001; Q1 - first quartile; Q3 - third quartile. Direct, indirect and total effects are exponentiated. | | | | | | | |

# Table S6. Sensitivity analysis. Results of the Cox proportional hazards models of associations between build environment assessments and all-cause mortality across men and women aged 65+, Polish part of the COURAGE in Europe study, after excluding individuals who died between 2011 and 2013 (N = 1166). Weighted data.

|  |  | **MEN** | | | **WOMEN** | | |
| --- | --- | --- | --- | --- | --- | --- | --- |
|  |  | Slow mobility users | | Fast mobility users | Slow mobility users | | Fast mobility users |
|  | Model | Walkers | Cyclists |  | Walkers | Cyclists |  |
| **COURAGE Built Environment Self-Reported Questionnaire (CBE-SR)** | | | | | | | |
| Usability of the neighborhood environment | I | 1.00 [0.99;1.01] | 0.99 [0.98;1.01] | 0.99 [0.98;1.01] | 0.99 [0.98;1.01] | 1.01 [0.99;1.03] | 1.00 [0.98;1.02] |
|  | II | 1.00 [0.98;1.01] | 1.00 [0.98;1.02] | 0.97 [0.92;1.02] | 1.00 [0.99;1.01] | 1.04 [0.98;1.09] | 1.01 [0.99;1.03] |
|  | III | 0.99 [0.98;1.00] | 1.00 [0.97;1.03] | 0.96 [0.92;1.01] | 1.00 [0.99;1.01] | 1.07 [0.97;1.17] | 1.00 [0.98;1.02] |
| Hindrance of walkable environment  (*reversed*) | I | 0.99 [0.98;1.01] | 0.99 [0.98;1.01] | 0.99 [0.97;1.02] | 1.00 [0.99;1.01] | 0.99 [0.97;1.01] | 0.99 [0.98;1.01] |
|  | II | 0.99* [0.98;0.99] | 0.99 [0.98;1.02] | 0.99 [0.95;1.02] | 0.99 [0.99;1.01] | 0.96*** [0.92;0.99] | 0.99 [0.97;1.02] |
|  | III | 0.98*** [0.97;0.99] | 0.99 [0.97;1.01] | 0.95 [0.91;1.00] | 0.99 [0.98;1.01] | 0.86 [0.74;0.99] | 0.98 [0.95;1.01] |
| Easiness of use of public buildings, places and facilities | I | 0.99 [0.99;1.01] | 0.98 [0.97;1.01] | 0.99 [0.97;1.01] | 0.99 [0.99;1.01] | 1.01 [0.99;1.04] | 0.99 [0.98;1.01] |
|  | II | 1.00 [0.99;1.02] | 0.98 [0.96;1.01] | 0.99 [0.91;1.08] | 1.00 [0.99;1.01] | 1.01 [0.98;1.03] | 1.02 [0.99;1.05] |
|  | III | 0.99 [0.98;1.01] | 0.98 [0.96;1.01] | 0.98 [0.94;1.04] | 1.00 [0.99;1.01] | 1.02 [0.99;1.05] | 1.00 [0.96;1.05] |
| Usability of the living place | I | 1.00 [0.99;1.02] | 0.98 [0.96;1.01] | 0.99 [0.97;1.01] | 1.00 [0.99;1.01] | 0.99 [0.97;1.02] | 0.99 [0.98;1.01] |
|  | II | 1.01* [1.00;1.03] | 0.98 [0.96;1.01] | 1.01 [0.97;1.05] | 1.01 [0.99;1.02] | 0.99 [0.96;1.02] | 0.99 [0.98;1.02] |
|  | III | 1.01 [0.99;1.02] | 0.98 [0.95;1.01] | 1.01 [0.94;1.08] | 1.01 [0.99;1.02] | 0.98 [0.95;1.02] | 0.98 [0.95;1.02] |
| **COURAGE Built Environment Outdoor Checklist (CBE-OUT)** | | | | | | | |
| Streetscape | I | 0.98 [0.95;1.01] | 0.99 [0.96;1.04] | 1.01 [0.96;1.06] | 0.98 [0.96;1.01] | 0.96 [0.91;1.01] | 1.02 [0.97;1.06] |
|  | II | 0.98 [0.95;1.02] | 1.03 [0.98;1.08] | 1.00 [0.90;1.12] | 0.97* [0.95;0.99] | 0.96 [0.80;1.14] | 1.01 [0.97;1.07] |
|  | III | 0.99 [0.96;1.03] | 1.04 [0.99;1.09] | 1.01 [0.87;1.18] | 0.97* [0.95;0.99] | 0.93 [0.86;1.02] | 1.01 [0.96;1.07] |
| Walkways | I | 0.98 [0.96;1.01] | 0.97 [0.93;1.01] | 1.07*** [1.03;1.10] | 1.01 [0.98;1.04] | 1.02 [0.98;1.07] | 0.97 [0.93;1.01] |
|  | II | 0.97** [0.94;0.99] | 0.99 [0.96;1.04] | 1.03 [0.95;1.13] | 1.01 [0.99;1.05] | 1.11 [0.97;1.28] | 0.99 [0.94;1.04] |
|  | III | 0.96*** [0.93;0.98] | 1.01 [0.96;1.07] | 1.01 [0.91;1.12] | 1.01 [0.99;1.05] | 1.05 [0.95;1.16] | 1.01 [0.94;1.08] |
| Bikeways | I | 0.99 [0.98;1.01] | 0.99 [0.97;1.01] | 0.99 [0.98;1.02] | 1.00 [0.99;1.01] | 0.70*** [0.68;0.72] | 1.01 [0.98;1.03] |
|  | II | 0.99 [0.99;1.00] | 0.99 [0.96;1.02] | 1.04** [1.01;1.07] | 0.99 [0.99;1.01] | 0.69*** [0.65;0.72] | 1.00 [0.97;1.03] |
|  | III | 0.99 [0.99;1.01] | 0.98 [0.95;1.02] | 1.06** [1.02;1.09] | 0.99 [0.99;1.01] | 0.67*** [0.60;0.74] | 0.99 [0.95;1.05] |
| Street crossing/intersections | I | 0.99 [0.99;1.01] | 0.99* [0.97;0.99] | 0.99 [0.98;1.01] | 1.00 [0.99;1.01] | 1.01 [0.99;1.02] | 0.99 [0.98;1.01] |
|  | II | 0.99 [0.99;1.00] | 0.98 [0.97;1.01] | 0.95** [0.93;0.99] | 1.00 [0.99;1.01] | 0.99 [0.96;1.02] | 0.99 [0.97;1.02] |
|  | III | 0.99 [0.99;1.00] | 0.98 [0.96;1.01] | 0.91** [0.86;0.98] | 1.00 [0.99;1.01] | 0.97 [0.93;1.03] | 0.99 [0.97;1.03] |
| Parking facilities | I | 1.01 [0.99;1.03] | 1.01 [0.98;1.04] | 0.96 [0.90;1.03] | 1.01 [0.99;1.02] | 1.03 [0.99;1.07] | 0.99 [0.97;1.02] |
|  | II | 1.00 [0.98;1.03] | 1.03 [0.99;1.07] | 0.99 [0.89;1.11] | 1.01 [0.99;1.03] | 1.04 [0.93;1.17] | 1.00 [0.98;1.03] |
|  | III | 1.00 [0.98;1.03] | 1.03 [0.99;1.08] | 0.96 [0.81;1.14] | 1.01 [0.99;1.03] | 1.11 [0.88;1.41] | 0.99 [0.97;1.02] |
| Public facilities and features of the street | I | 0.99 [0.98;1.01] | 0.99 [0.97;1.02] | 1.03* [1.01;1.06] | 0.99 [0.98;1.01] | 0.99 [0.96;1.02] | 0.97 [0.94;1.01] |
|  | II | 0.99 [0.98;1.01] | 1.01 [0.98;1.05] | 0.98 [0.95;1.04] | 0.99 [0.97;1.00] | 1.03 [0.97;1.10] | 0.97 [0.93;1.01] |
|  | III | 1.01 [0.99;1.02] | 1.01 [0.97;1.05] | 0.98 [0.91;1.06] | 0.99 [0.97;1.01] | 1.03 [0.97;1.11] | 0.94** [0.91;0.98] |
| Land-use visible along the street/road | I | 0.98 [0.97;1.01] | 0.99 [0.97;1.02] | 1.01 [0.98;1.05] | 0.99 [0.97;1.01] | 0.99 [0.94;1.04] | 0.99 [0.96;1.02] |
|  | II | 0.98 [0.97;1.01] | 1.01 [0.96;1.07] | 0.99 [0.92;1.08] | 0.99 [0.97;1.01] | 0.99 [0.93;1.05] | 0.98 [0.93;1.04] |
|  | III | 0.99 [0.97;1.01] | 1.01 [0.96;1.06] | 0.97 [0.86;1.08] | 0.99 [0.97;1.01] | 1.02 [0.96;1.08] | 0.97 [0.90;1.03] |
| Site decay/urban blight | I | 1.01 [0.99;1.03] | 1.04  [0.98;1.10] | 1.01 [0.97;1.06] | 0.99 [0.98;1.01] | 1.06 [0.99;1.14] | 0.99 [0.95;1.04] |
|  | II | 0.99 [0.98;1.02] | 1.02 [0.94;1.11] | 0.97 [0.88;1.06] | 0.99 [0.97;1.02] | 1.16 [0.92;1.46] | 0.98 [0.94;1.03] |
|  | III | 0.99 [0.98;1.02] | 1.05 [0.96;1.15] | 0.98 [0.87;1.09] | 0.99 [0.97;1.02] | 1.18 [0.86;1.61] | 0.99 [0.93;1.07] |
| Note: Model I - unadjusted Model II – adjusted for age, marital status, level of education, place of living, BMI, physical activity, grip strength, total number of diseases, visual difficulties, ADL, IADL, perceived safety on the street; Model III – additionally adjusted for loneliness, level of social networks social support, and formal participation; ; *p < 0.05; **p < 0.01; ***p < 0.001 | | | | | | | |
|  |  |  |  |  |  |  |  |
|  | | | | | | | |

#

# Table S7. Sensitivity analysis. Results of the Cox proportional hazards models of associations between mobility mode and all-cause mortality across men and women aged 65+, Polish part of the COURAGE in Europe study, after excluding individuals who died between 2011 and 2013 (N = 1166). Weighted data.

|  | **MEN** | | | **WOMEN** | | |
| --- | --- | --- | --- | --- | --- | --- |
|  | Slow mobility users | | Fast mobility users | Slow mobility users | | Fast mobility users |
| Model | Walkers | Cyclists |  | Walkers | Cyclists |  |
| I | ref. | 0.93 [0.58;1.50] | 0.97 [0.56;1.69] | ref. | 0.58 [0.31;1.12] | 0.76 [0.47;1.26] |
| II | ref. | 0.73 [0.43;1.24] | 0.92 [0.51;1.65] | ref. | 0.97 [0.43;2.21] | 0.85 [0.48;1.49] |
| III | ref. | 0.84 [0.49;1.45] | 1.00 [0.49;1.45] | ref. | 0.95 [0.41;2.18] | 0.89 [0.51;1.56] |

Note: Model I - unadjusted, adjusted for age, marital status, level of education, place of living, BMI, physical activity, grip strength, total number of diseases, visual difficulties, ADL, IADL, perceived safety on the street; Model III – additionally adjusted for loneliness, level of social networks social support, and formal participation;
